# Bioengineering tools to speed up the discovery and preclinical testing of vaccines for SARS-CoV-2 and therapeutic agents for COVID-19

**DOI:** 10.7150/thno.47406

**Published:** 2020-05-27

**Authors:** Manuela Teresa Raimondi, Francesca Donnaloja, Bianca Barzaghini, Alberto Bocconi, Claudio Conci, Valentina Parodi, Emanuela Jacchetti, Stephana Carelli

**Affiliations:** 1 Department of Chemistry, Materials and Chemical Engineering G. Natta, Politecnico di Milano, Milano, Italy.; 2 Pediatric Clinical Research Center “Fondazione Romeo ed Enrica Invernizzi”, Department of Biomedical and Clinical Sciences L. Sacco, University of Milano, Italy.

**Keywords:** Coronavirus, bioengineering, target, antiviral, vaccine, preclinical testing

## Abstract

This review provides an update for the international research community on the cell modeling tools that could accelerate the understanding of SARS-CoV-2 infection mechanisms and could thus speed up the development of vaccines and therapeutic agents against COVID-19.

Many bioengineering groups are actively developing frontier tools that are capable of providing realistic three-dimensional (3D) models for biological research, including cell culture scaffolds, microfluidic chambers for the culture of tissue equivalents and organoids, and implantable windows for intravital imaging.

Here, we review the most innovative study models based on these bioengineering tools in the context of virology and vaccinology. To make it easier for scientists working on SARS-CoV-2 to identify and apply specific tools, we discuss how they could accelerate the discovery and preclinical development of antiviral drugs and vaccines, compared to conventional models.

## Introduction

As of May 8, 2020, the 2019 novel coronavirus SARS-CoV-2 was responsible in 215 countries for 3,767,744 infections and 259,593 deaths from the related disease, defined as COronaVIrus Disease 2019 (COVID-19) 1. The human population has no immunity to SARS-CoV-2 because this virus had never previously infected humans before its initial outbreak in December 2019. Until now, no drugs or biologics have been proven to be effective for the prevention or treatment of COVID-19. To deal with this pandemic, apart from non-pharmaceutical containment measures including quarantine and maintenance of social distance, possible interventions consist of the repurposing of existing pharmaceutical products, along with the development of new products and vaccines 2-4.

The development of a radically new pharmaceutical product, either traditional, or biological such as an antibody targeting a SARS-CoV-2 receptor, is subject to regulatory approval for use in humans based on three steps: 1) lab discovery *in vitro* on cell cultures; 2) preclinical testing *in vivo* in animals to confirm the mechanism of action and to measure the range of effective dosages and tolerability; and 3) clinical testing in patients to define the safe dosage and to confirm the efficacy and possible interactions. This process involves serious challenges both related to the efficiency, for example the development of a human vaccine from concept to licensing takes at least 15 years 5, and also to the effectiveness. Drug development currently involves a 99.9% overall failure rate, of which 96.4% is due to failure in the preclinical testing phase, meaning that the drug efficacy measured *in vitro* is almost never confirmed in animals 6.

The main issue is that the most widespread technology currently used to test therapeutic agents *in vitro* is obsolete: often it consists of a two-dimensional (2D) polystyrene culture dish, in which a single cell population is cultured on the bottom, the drug to be tested is added to the culture medium, and the expected modulation in specific processes or targets is measured.

However, in these simplified *in vitro* culture conditions, the drug elicits a cell response that is not representative of the *in vivo* response, which is based on the cell interactions that occur a) in three-dimensional (3D) non-flat environments, and b) within an heterogeneous cell population which is never limited to the cell population primarily addressed by the drug.

Another very serious issue is the unethical slaughter of lab animals, which more effective *in vitro* methods would replace, at least in part. Moreover, the methods adopted for monitoring the drug effects in animals are also obsolete, whereas new miniaturized intravital imaging techniques are now available that could greatly refine *in vivo* observations, even in terms of the temporal evolution in the same animal, thus reducing the number of animals sacrificed by 80-90%.

There are many on-going clinical trials evaluating potential vaccines and treatments for COVID-19, which cannot be accelerated without putting the safety of patients at risk. Instead, both the *in vitro* and *in vivo* preclinical phases of drug development could be accelerated by replacing some of the current unrepresentative and obsolete study models, with new ones based on improved modeling tools. For example, many groups have developed 3D static, microfluidic and intravital imaging models of viral infection and relevant therapeutics using new high-performance experimental devices that are user-friendly for operators and enable live organoids to be cultured and examined in 3D with high-resolution imaging.

Here, we review the published work based on these modeling tools in the specific fields of virology and pharmacology.

## Recent developments

### *In silico* discovery of drug targets for SARS-CoV-2 and prediction of its pathogenic mechanisms

SARS-CoV-2 infection can lead to COVID-19 infection, which causes massive damage to the pulmonary cells 7-11 due to a cytokine storm, *i.e.* an acute secretion of inflammatory signals by innate monocytes in response to the infection, which causes hyper inflammation. The WHO 12 and Clinicaltrials.gov database currently report many active trials on COVID-19 patients of drugs that have already been licensed for other infectious and inflammatory diseases. These include anti-malarial drugs, such as chloroquine and its derivative hydroxychloroquine, large spectrum antivirals (inhibitors of endonucleases, nucleosides, protease inhibitors, and other antiretrovirals), interferons, corticosteroids, immune suppressants, and anticoagulants.

**Figure 1:**
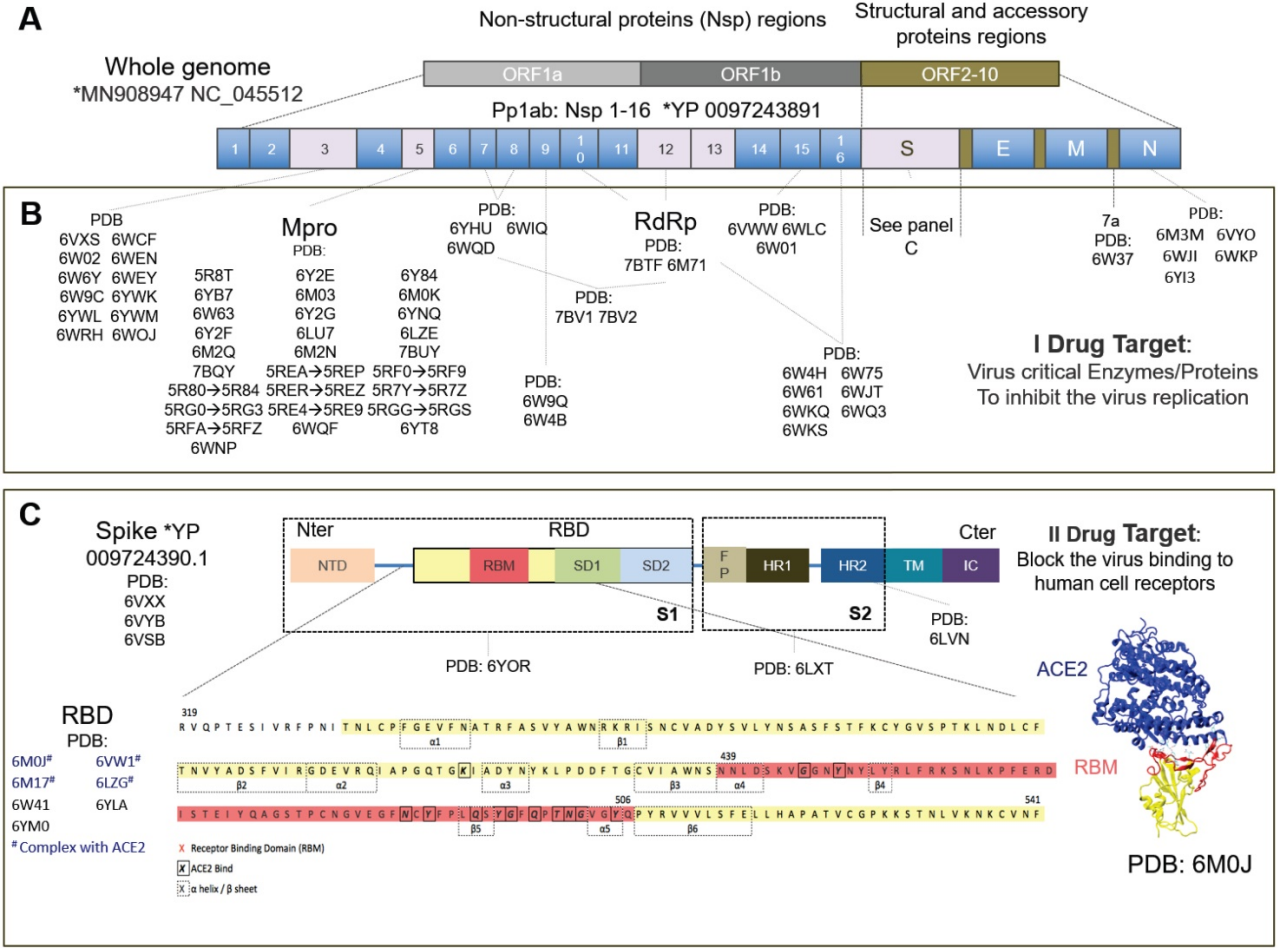
** Drug targets specific to the SARS-CoV-2 virus. A)** The SARS-CoV-2 whole genome consists of three main open reading frames (ORF): ORF1a, ORF1b and ORF2-10. The most frequent targets for therapies are reported in gray with their PDB codes. **B)** Some proteins encoded in ORF1a and ORF1b represent the primary I potential drug target. Their inhibition blocks the viral RNA synthesis and replication. Pp1ab encodes for 16 non-structural proteins (NSPs) including the papain-like protease (PLpro) in the Nsp3region, main protease or C-like protease (Mpro or 3CLpro), ADP ribose phosphatase (ADRP), RNA-dependent RNA polymerase (RdRp) and helicase. ORF2-10 encodes for accessory proteins (*e.g.* 7a) and the structural and accessory proteins, spike protein (S), envelope protein (E), membrane protein (M), and nucleocapsid protein (N). **C)** The spike protein is the most studied secondary II potential drug target. The virus cannot bind the cell receptors if the spike protein is inhibited 14. The spike protein has been studied the most, has been solved by electron microscopy (EM) and consists of: N-ter domain (NTD); receptor binding domain (RBD) consisting of receptor binding motif (RBM), subdomain 1 (SD1) and subdomain 2 (SD2); fusion peptide (FP); heptad repeat 1 (HR1); heptad repeat 2 (HR2); transmembrane region (TM) and intracellular domain (IC). The most studied RBD domain is RBM (in red), the domain mainly involved in host-cell interaction 13. RBM is believed to bind mainly with the ACE2 human cell receptor and, therefore, most of the relevant PDB codes, of which 6M0J, includes the RBD+ACE2 complex. The complete amino acid sequence for RBD is shown at the bottom of the figure. Amino acids corresponding to RBM are in red, the beta-sheet and alpha-helix structures are inside the dotted boxes. The contact residues at the RBD/ACE2 interface 14 are shown in bold squares. *https://www.ncbi.nlm.nih.gov/genbank/sars-cov-2-seqs/.

In parallel, many groups are repurposing known compounds and are developing new vaccines 3 and therapeutic solutions 4 to specifically target the SARS-CoV-2 virus. The drug targets are primarily viral proteins, most of which have been resolved and are available on the protein data bank (PDB). Much progress has been made in the structural biology of SARS-CoV-2. Many structural and non-structural proteins have been determined experimentally by cryo-electron microscopy (cryo-EM) and X-ray crystallography. The proteins specific to SARS-CoV-2 that have already been determined experimentally are shown in Figure 1.

Drug therapies may act either a) on the host cells, affecting the specific receptor or restoring the innate immunity of the cells, or b) on the virus, by blocking the virus functions (I target) or acting on the virus structural proteins (II target). The primary (I) drug target is represented by several non-structural viral proteins (NSPs) whose inhibition blocks the viral RNA synthesis and replication 13. These include the papain-like protease (PLpro) in the Nsp3 region, the main protease (Mpro) also known as C-like protease (3CLpro), the RNA-dependent RNA polymerase (RdRp) and the helicase.

The structural spike protein on the viral envelope is the most studied protein of the secondary (II) drug targets 13. The virus cannot bind to the host cell receptors, fuse with cell membranes and enter cells, if the spike protein is inhibited, and thus it has also already been solved by electron microscopy 14. This comprises a receptor binding domain (RBD), mainly involved in the host-cell interaction, incorporating a sub-domain called a receptor binding motif (RBM) believed to bind, similarly to SARS-CoV, mainly with the human angiotensin-converting enzyme 2 (ACE2) cell receptor, to gain entry inside cells 15. The ACE2 receptor was discovered in 2000 and is expressed above all in the lungs, heart, kidneys, gastrointestinal tract, testis and other tissues or organs 16. Most drugs against SARS-CoV-2 investigated today therefore target the complex RBD+ACE2 17-19.

Solved SARS-CoV-2 molecular structures have been used to set-up docking analyses and molecular dynamics simulations that predict the affinity of chemical binding of these structures with known compounds. This *in silico* modeling technique has been used intensely for drug screening in virtual libraries. Table 1 shows the latest published results in terms of drugs predicted to bind with SARS-CoV-2 targets. It is worth noting that most of the *in silico* drug docking analyses listed in Table 1 use the homology modelling strategy to predict the structures of the target proteins that have not yet been experimentally resolved 20-25. The homology modelling approach predicts the atomic-resolution model of a target protein based on the structure of the protein chosen as a template according to the genome sequence identity 26. The genome of SARS-CoV-2 showed a high homology with the SARS virus, which makes it a reliable template 13.

The sequence alignment with the previous SARS-like viruses have also been applied to perform the virus evolutionary analysis 27, or to highlight particular features of the SARS-CoV-2 virus (Table 2). Several studies have compared SARS-CoV-2 with SARS in terms of interaction of the spike protein with the host cell receptor. Structural- and free-binding energy-based analyses have revealed accurate details of SARS-CoV-2. These observations may explain the aggressiveness of SARS-CoV-2, at least in part 17-19,28.

A similar approach has also been taken to explore the efficiency of the binding of the proteins encoded by different human ACE2 allelic variants with the SARS‐CoV‐2 spike protein. From the binding analysis, two variants showed variations in the orientation of the amino acid side chain, altering the intramolecular interactions and the complex formation. This aspect may represent partial intrinsic susceptibility or resistance to the SARS‐CoV‐2 infection 29.

### 3D cell models to improve pre-clinical *in vitro* studies

Once discovered *in silico*, the drug has to be tested *in vitro* and *in vivo* in experimental models of viral infection.

**Figure 2:**
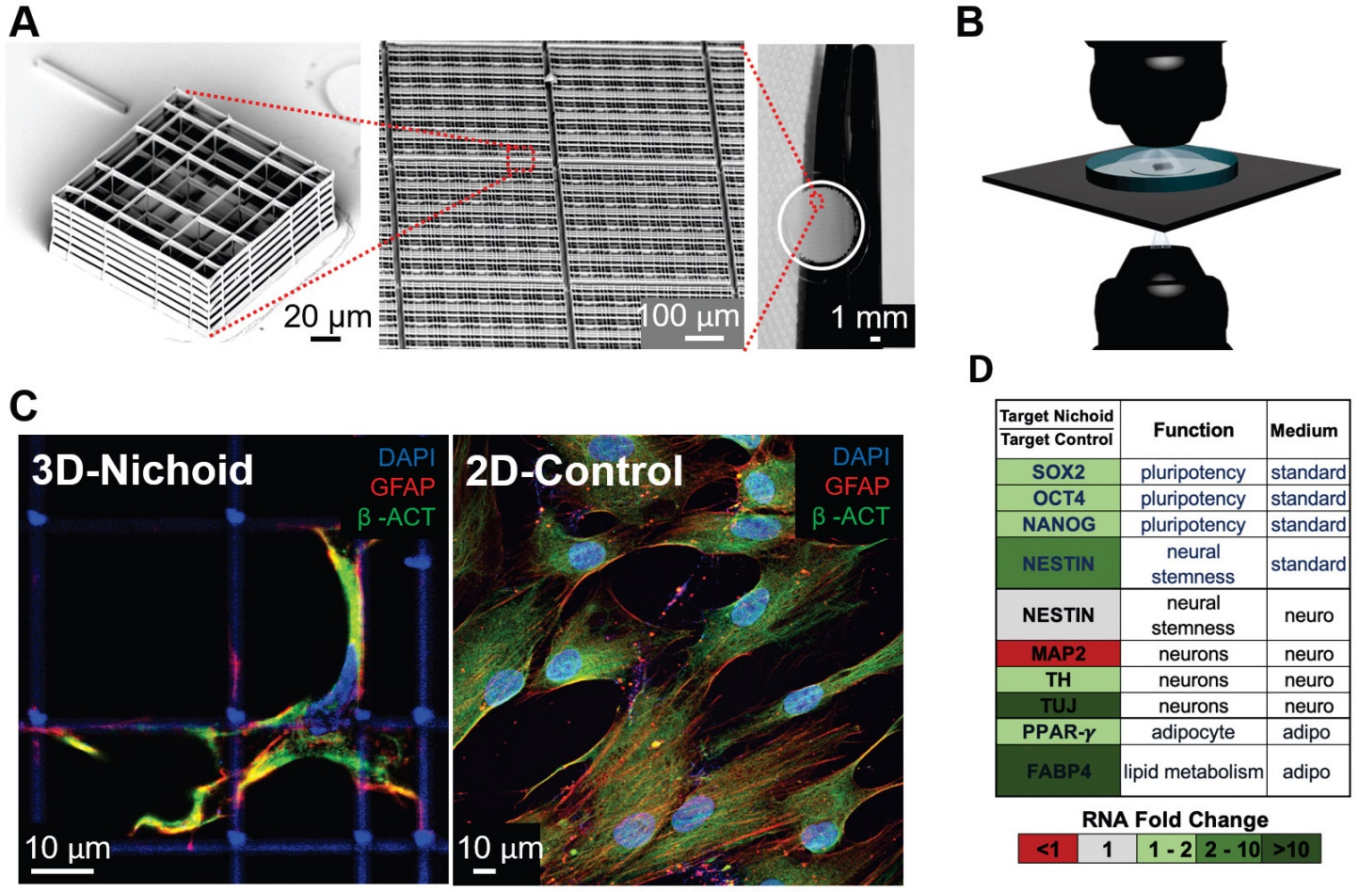
** Micro-fabricated “Nichoid” scaffold for 3D cell culture. A)** Nichoid architecture. From left to right: scanning electron microscopy (SEM) image of a Nichoid single-module; SEM image of up-scaled repetitive matrixes of Nichoids; picture of 50 mm^2^ micro patterned glass coverslip with Nichoids. Nichoid culture 3D-substrates are produced via two-photon laser polymerization of SZ2080 negative photoresist with near-infrared exposure following a CAD geometry. **B)** Possible microscopy configurations to use on Nichoid culture substrates. Thanks to the device's versatility, this scaffold enables optical accessibility both in transmission and in reflection modality. **C)** Immunofluorescence images obtained via confocal fluorescence microscopy of GFAP (red), β-ACTIN (green) and DNA (blue), in human Adipose derived Stem Cells (hADSCs) after a 7-day expansion inside the Nichoid and in standard conditions (2D-Control). The images demonstrate that nuclear morphology and protein organization and localization (both β-ACTIN and GFAP) differed between the two culture systems. Cytoskeletal markers merge (yellow signal) into cellular protrusions inside Nichoids, while a poor signal appears in flat conditions. **D)** Real-time PCR analysis of specific gene targets shows a significant gene expression difference (up-regulated in green, and down-regulated in red) between Nichoid and Control conditions, both using a standard culture medium and also using a culture medium that induces adipogenic or neural differentiation.

Our aim was to include any advanced study model developed for virology and vaccinology since the SARS coronavirus outbreak of 2003 and we analyzed the literature pertaining to the last two decades. We found 40 papers, 75% of which were published in the last five years. 3D cell models are generally based on cell aggregates or organoids, cultured within a matrix or scaffold that provide a 3D environment for cell growth (Figure 2). Organoids are usually obtained starting with pluripotent stem cells (most frequently iPS), while engineered tissues are mainly derived from multipotent or unipotent cells.

To date, only five very recent studies have addressed SARS-CoV-2 with such an innovative approach: specifically they replicate the SARS-CoV-2 infection in models of the human airway epithelium 30-32, human liver organoids 33 and in engineered human kidney and vessel organoids 34. In addition, three of the above studies include therapeutic agents to inhibit SARS-CoV-2 infection: clinical-grade human recombinant soluble angiotensin converting enzyme 2 (hrsACE2) 34, remdesivir also in association with diltiazdem 31 and β-D-N4-hydroxycytidine 32. Table 3 reports the findings of the published 3D cell models used to study viral infections, and Table 4 reports those that also include the effects of an antiviral therapy.

Most studies on viruses other than SARS-CoV-2 consist in modeling respiratory infections diffused in an engineered human pseudostratified airway epithelium 35-44. In these models, the cellular constructs are based on primary epithelial cells embedded in a matrix of biological origin replicating the basal membrane, and are grown to full differentiation, preferably at the air-liquid interface, into an organoid with basal, secretory, and multi-ciliated cells. Most studies also use specific control cell lines cultured in monolayer, for example derived from lung and intestinal adenocarcinoma epithelial cells 32, 45-46, or from lung primary fibroblasts and epithelial cells 40. A comparison of the results from 3D models with monolayer cultures highlights the following significant advantages of 3D cell models in replicating conditions observed *in vivo*.

#### 1) Cell morphology, virus penetration and cell damage

These parameters can be imaged and are replicated similarly to *in-vivo* in 3D cell and organoid models, unlike in cell monolayers. For example, cells in 3D engineered airway epithelium infected with SARS-CoV-2 showed apical-apical polarity, which is known to restrict the infection to the airway lumen *in vivo* 30, and reproduced infection peaks and significant morphological alterations earlier in nasal cells compared to bronchial cells 32. Cells in liver organoids infected by SARS-CoV-2 33 and cells in intestinal organoids infected by MERS-CoV 46 had syncytia and membrane fusions as observed in mice *in vivo* models. When infected by the respiratory syncytial virus (RSV), lung organoids had a morphological structure and infection features matching human fetal lung tissue 35, and airway epithelium organoids had massive epithelial modifications as in mature tissue infection *in vivo* 36. In a 3D engineered airway epithelium, cell morphology was close to *in-vivo* characteristics*,* with a reorganization of cytoskeletal components, an abundance of ciliated cells 38, and a mucociliary layer 41, with surfactant and mucus often produced 37. Here, the H7N9 influenza virus infected both ciliated and non-ciliated cells, causing cilia and tight junction damage seen *in vivo* 42. A 3D engineered liver model reproduced and demonstrated greater physiological hepatocyte morphology, polarity, and apical/basal adhesion to ECM compared to a classical cell monolayer 47.

#### 2) Susceptibility to viral infection

3D cell and organoid models are significantly more permissive to viral infection, compared to cell monolayers. For example, when infected by SARS-CoV-2, the peak infection in 3D-engineered human airway epithelium was reached 30% faster 31; also, when infected by avian influenza virus H7N9, viral proliferation was faster 42. In addition, propagation of human rhinovirus C and bocavirus was two and three orders of magnitude greater, respectively 41. In airway epithelium organoids infected with avian influenza virus such as H1N1, the viral proliferation was faster and with a time-order equivalent to the *in vivo* proliferation time 38. MERS-CoV was shown to infect intestinal organoids more than epithelial monolayers 46. In a model of HIV-1 infection of primary human CD4^+^ T-lymphocytes, 3D collagen culture, unlike suspension conditions, promoted cell-associated HIV-1 transmission, consistent with spread mechanisms described *in vivo* 48. Human intestinal organoids infected with rotavirus displayed a 10,000-fold increase in genomic viral RNA than cells infected in monolayer 49. Hepatitis B replicated one order of magnitude faster in a 3D-engineered liver model, compared to a cell monolayer 47.

#### 3) Cell expression of virus receptors, antiviral genes, antigens to viruses and inflammatory markers

The expression of all these markers is more similar in time and space to *in-vivo* values*,* in 3D cell and organoid models, compared to cell monolayers. For example, a 3D-engineered airway epithelium infected by SARS-CoV-2 showed different expression levels of immune-response genes (type I and type III interferons and a subset of genes associated with the NF-kB and TNFα pathways) between nasal cells (with strong upregulation at 24 hours post infection) and bronchial cells (with low expression) 31. Cells in liver organoids expressed the ACE2 receptor, thus enabling SARS-CoV-2 infection, unlike in the mouse model 33. When infected by the respiratory syncytial virus (RSV), lung organoids had gene and marker expression (primarily the mesenchymal markers PDGFR-α, PDGFR-β, α-SMA, vimentin and CD90) that matched infected human fetal lung tissue 35, while airway epithelium organoids expressed levels of genes involved in migration (KRT16, KRT6b), interferon signaling (IL1RN, IFI44L) and viral response (MX2, OAS1), comparable to *in vivo* values 36.

In 3D-engineered airway epithelium, expression of inflammatory cytokines (aquaporin-5 and cytokeratin-14) was two-fold higher than monolayers when infected with avian influenza viruses H1N1 and H3N2 37, and two orders of magnitude higher (for IL-6 and IL-8) when infected with human rhinovirus C and human bocavirus 41, closely recapitulating the immune response seen *in vivo*. In a model of stratified airway epithelium infected by the andes-hantavirus, long-term culture at seven days showed a switch in immune response from anti-viral (transient interferon) to antinflammatory (cytokines and VEGF-A), as occurs *in-vivo* 40. Hepatitis B induced a two-fold higher expression of specific antigens (HBsAg) in a 3D-engineered liver model, compared to cell monolayers 47.

#### 4) Sensitivity to antiviral drugs

In 3D models, the sensitivity to antiviral drugs is significantly lower and more similar to *in vivo* than cell monolayer observations. For example, the efficacy of clinical-grade soluble human ACE2 in reducing SARS-CoV-2 infection in both human capillary organoids and kidney organoids was one order of magnitude lower than in monolayers 34. In a 3D-engineered airway epithelium infected with the H7N9 influenza virus and treated with recombinant human interferons α2b and λ1, the expression of interferon-stimulated antiviral genes was significantly reduced, and the level of gene expression induced by the two agents was reversed, consistently with data *in vivo*, compared to monolayers 42. Human intestinal organoids infected by human rotavirus, compared to the simple cell line model in monolayers, were twice less sensitive to interferon α and ribavirin, with a range of effective doses comparable to that administered in *in vivo* models 49.

The response to the antiviral drug entecavir in a 3D human engineered liver model infected by hepatitis B was more similar to *in vivo*, compared to the cell line model 47. In conventional cell monolayers, the response of herpes simplex infection to antivirals is unrealistically fast with 100% reactivation. For herpes simplex 1, virus reactivation after exposure to interferon α was reduced five-fold in infected 3D brain organoids compared to cell monolayers, as reported in animal models 50. In addition, in hydrogels made of acrylated hyaluronic acid and loaded with fibroblasts, the time for cell recovery and reaggregation after infection and exposure to the antiviral drug, acyclovir was three-fold greater than in cell monolayers, similarly to *in vivo* 51.

The evidence outlined above shows that 3D cells and organoid models consistently develop properties that are able to determine the *in vivo*-like dynamics of infection by pathogenic viruses. They thus may represent a valuable tool towards a more rapid assessment of infectivity to humans of emerging respiratory viruses such as the SARS-CoV-2, and of relevant drug responses.

From a technological point of view, there are two principal limitations of the previously analyzed 3D models compared to conventional monolayer models, which are essentially related to the 3D scaffolds. The current scaffolds are mainly represented by either hydrogels 52-53 or solid ECM analogues 54, which are placed on the bottom of culture plates or in trans-well inserts, resulting in constructs which are a few millimeters thick and cannot be examined under optical microscopy. The second problem in current 3D scaffolds is the very limited control over the culture time of some physical and chemical properties known to profoundly affect cell behavior, such as biodegradation with the release of various solutes, and mechanical properties such as stiffness, which influence cell adhesion and migration 54. To overcome all these limitations, many groups of researchers are developing miniaturized 3D scaffolds by a cutting-edge fabrication technique called two-photon laser polymerization 55.

For example, our group has developed an innovative 3D micro-scaffold called the Nichoid (Figure 2), which is optically and physically accessible to the cells cultured inside, over time, and to all the existing biological assays. The Nichoid is a miniaturized rigid, transparent, ultra-precise 3D grid fabricated in a biocompatible resin 56-58. This scaffold is up to 100 microns thick, thus truly 3D for cells but 100% optically accessible to high-resolution fluorescence diagnostics. By mimicking the 3D stiffness of a physiological cell niche 59, the Nichoid can guide the self-organization of several types of stem and progenitor cells 60-61, including embryonic stem cells 62 and mesenchymal stem cells (MSC) 63. It also maintains their phenotype in expansion culture, by regulating hundreds of genes involved in mechanotransduction processes such as cell migration, cytoskeletal organization, and membrane plasticity 64. The Nichoid could thus provide an effective culture environment to test mechanobiological effects such as the overexpression of interferon-induced transmembrane proteins that can rigidify the cell membrane, thus markedly inhibiting viral membrane fusion and entry. It could thus be used for screening candidate antiviral drugs and vaccines based on the targeting coronavirus spike protein. We also observed that the Nichoid potentiates the therapeutic efficacy of stem cells expanded inside 65. This substrate is a powerful candidate to maintain the safety and function of expanded MSC 63, which is a new therapy already being tested in clinical trials to treat post-COVID-19 pulmonary fibrosis 66.

### Bioreactor-perfused cell models

**Figure 3:**
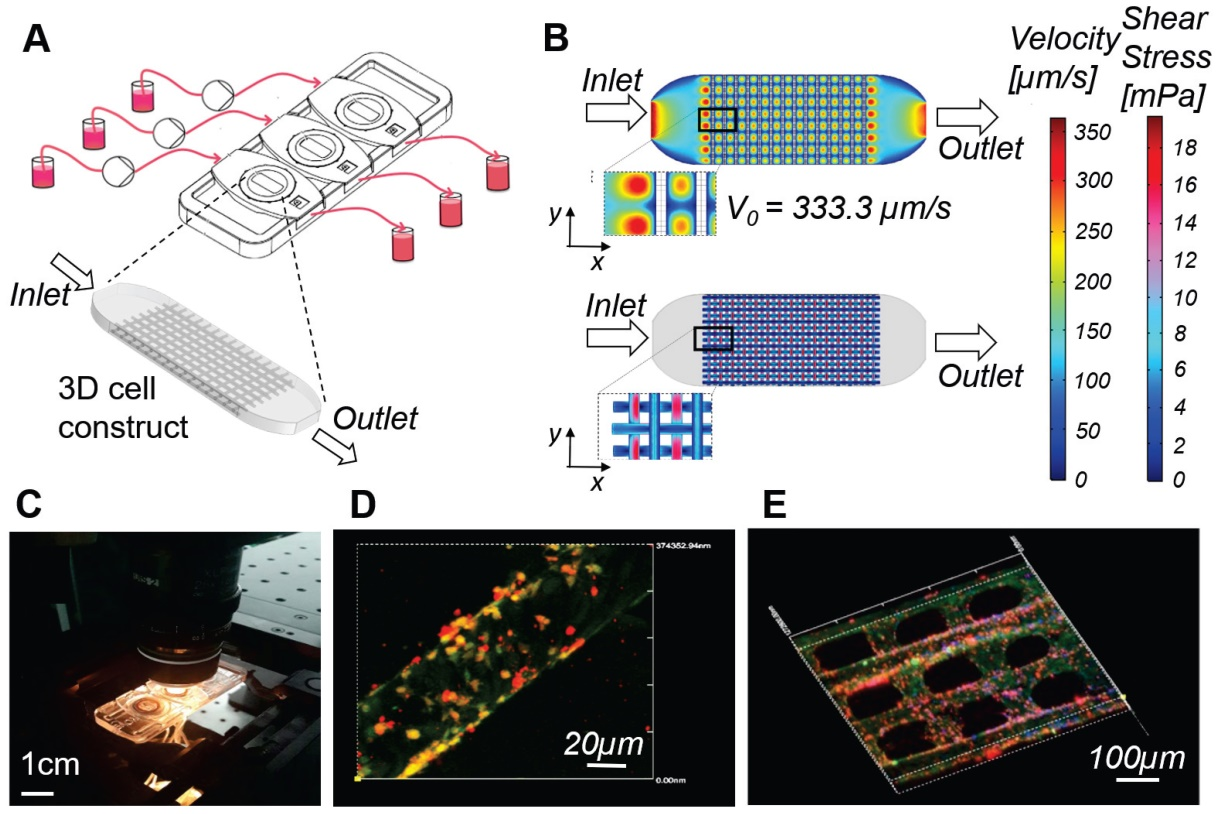
** Millifluidic optically accessible bioreactor (MOAB) for perfused culture of 3D cell constructs. A)** The system is composed of, from left to right: culture medium reservoirs, microfluidic pumps, bioreactor chamber and reservoirs for medium collection. CAD model of the 3D cell construct or organoid, cultured in the culture chambers, used for numerical simulations. **B)** Results of the computational fluid dynamics (CFD) simulations for a single culture chamber of the system. Top: fluid velocity (modulus of the velocity vector) is mapped on the whole culture chamber. Bottom: wall shear stress (WSS) is computed and mapped at the cell-culture medium interface. **C)** Photo of the MOAB showing the three independent chambers connected to the perfusion chamber by oxygenator tubes; the device is placed on a confocal microscope connected to a CPU allowing real time imaging of a perfused lymph-node-on-a-chip model, for the development and testing of vaccines and agents for cancer immune-therapy. **D)** Fibroblast reticular cells (yellow) are seeded on the 3D fiber scaffold. Dendritic cells introduced in suspension during culture medium flow adhere to the fibroblast reticular cells, migrate to the scaffold, and are activated to express the adhesion receptor ICAM-1 (red). **E)** Antigen-specific T cells (cyan) introduced with culture medium flow tend to adhere to dendritic cells expressing ICAM-1 (red) while crawling on the scaffold.

In bioreactor culture systems, the cellular constructs or organoids are hosted in culture chambers, which are connected to a hydraulic circuit that perfuses the culture medium with a pump (Figure 3). Microfluidic systems are the miniaturized version of bioreactors, generally consisting in thin transparent devices, similar to microscopy slides, with integrated micro-channels that can host cell-loaded hydrogels or suspensions. Table 5 shows the viral infection models that have been developed using bioreactor devices. The literature reports a pioneering model of SARS-CoV infection in a 3D bioreactor-engineered respiratory epithelium 67-68 and several other more recent models of infection based on different types of viruses. These studies demonstrate the following significant advantages of perfused culture in replicating conditions seen *in vivo*, compared to non-perfused culture.

#### 1) Perfusion increases the degree of cellular differentiation

For example, a 3D bioreactor-engineered respiratory epithelium infected with SARS-CoV was found to more closely resemble normal human tissue in terms of cell apical polarity, essential to viral cell-cell transport, compared to non-perfused culture 67-68. A bioreactor-engineered liver model of hepatitis B infection recapitulated the functional hepatic microarchitecture and complete cell polarization, which is critical for the cells' susceptibility to infection, compared to non-perfused organoids which show de-differentiation over 10-13 days 70. A bioreactor-engineered model of neuronal tissue based on neural progenitors showed some features of the human trigeminal ganglia, the site of latency of the varicella-zoster virus, not seen in non-perfused cultures 68-69.

#### 2) Perfusion extends cell viability, cell metabolism and functional stability

A 3D bioreactor-engineered respiratory epithelium infected with SARS-CoV remained viable three-times longer than non-perfused controls 67-68. A bioreactor-engineered liver model of hepatitis B infection was metabolically and functionally stable for at least 40 days, *i.e.* four times longer than conventional non-perfused cultures 70. A bioreactor-engineered model of neuronal tissue infected by the varicella-zoster virus was successfully maintained in culture for 180 days, compared to the limit of a few days for primary human ganglia in non-perfused culture 68-69.

#### 3) Perfusion increases the infection efficiency for longer incubation times

In a microfluidic model of fibroblast infection by the adenovirus, a 50% higher infection efficiency was maintained at a lower multiplicity of infection (the minimum virus/cell ratio able to cause infection) for longer incubation times, with respect to non-perfused controls 71. A bioreactor-engineered liver model of hepatitis B infection led to a 10,000-fold lower multiplicity of infection, compared to non-perfused culture 70. A bioreactor-engineered model of neuronal tissue infected by the varicella-zoster virus maintained persistent viral infection for 90 days, which is not possible with non-perfused culture 68-69.

#### 4) Perfusion increases the level and stability of cell marker expression

In a 3D bioreactor-engineered respiratory epithelium infected with SARS-CoV, the antibody response to SARS-CoV-specific spike and nucleocapsid glycoproteins was two-fold, compared to non-perfused culture 67-68. A bioreactor-engineered liver model of hepatitis B infection maintained stable expression levels of receptors of innate immune cells and downstream effectors, compared to non-perfused cultures 70.

The evidence above therefore shows that perfused models enable infection studies to be extended even up to several months, on stable cell cultures of highly differentiated cells, with a higher infection efficiency and lower multiplicity of infection, compared to non-perfused models. This makes it potentially feasible to extend the study of the life cycle and genetic stability of new viruses such as SARS-CoV-2 to *in vitro* systems, despite being derived from minimal doses from patients, and to test sequential antiviral drug treatments 70. Most importantly, perfused cell models make it feasible to reproduce dynamic interactions between tissue-resident cells and circulating cells, including the response to a pathogen of innate and adaptive immune cells and thus, to extend the testing of vaccines to *in vitro* models.

A first step forward in this regard is a recent microfluidic-based model of a lymph node 72. Here, the stroma was modeled with adherent murine tumor dendritic cells, and CD4^+^ and CD8^+^ T lymphocytes were put in suspension in the culture medium and recirculated interstitially within the engineered organ. This system selectively promoted the adhesion of antigen-specific T cells through affinity isolation by serial cell contacts, with fluid-induced shear stresses maintained in the range 0.1-1 dyn/cm^2^. In contrast to non-perfused cultures, this model was able to compare in parallel and in real time, the effect on T cell adhesion to dendritic cells of different treatments, inhibitors, activators and immunogens.

With regard to vaccine testing, bioreactors that host cm-sized 3D scaffolds such as the rotating wall vessel are able to provide tens of millions cells for confirmation assays such as PCR or FACS, as required by regulatory agencies. Their technological limitation, however, is that they are too thick to be accessed thoroughly for real time optical inspection of cell interactions 54,73. Microfluidic bioreactors, instead, are 2D and have micron-sized channels that are also fully inspectable in real time 74-75. However, they can host only a few thousand cells which are difficult to harvest and in any case would not be sufficient for robust confirmation assays 76.

To overcome these limitations, our group developed a millifluidic optically-accessible bioreactor (MOAB), which is a compromise between the two categories mentioned above. The MOAB (Figure 3) is a chambered microscope slide allowing the culture of fully 3D cell models with dimensions of up to a few millimeters, containing several million cells, under interstitial perfusion of the culture medium, with the infusion of cells in suspension and the therapeutic agents to be tested. The culture chambers are only 400 microns thick and enable full-thickness high-resolution fluorescence diagnostics, both in real time and post-cultivation 77-81. In *in vitro* tests, the MOAB led to a comparable cell response to biodrugs to the one seen in experimental animals, in specific fields characterized by slowly-developing diseases and slowly-developing healing induced by the therapeutic agent: stem cell therapy for neurodegeneration 82, gene therapy for muscular dystrophy 83, and chemotherapy for bone metastases 84.

By adding lymphocytes in suspension in the perfused medium (Figure 3), the MOAB easily extended to 3D a microfluidic model of lymphocytes instruction within a lymph node 72. Millifluidic bioreactors in fact have a high potential to speed up the discovery and testing of drugs and new vaccines for SARS-CoV-2 and are not limited to the interactions with the immune system. For example, they can successfully maintain human primary neurons infected by a virus in long-term culture for up to 180 days, compared to the limit of a few days in non-perfused culture 68-69. Millifluidic bioreactors can even be hydraulically connected in multiple units, to reproduce transport of neurotoxic agents through multiple body barriers pertaining to distant body compartments, for example from the gut endothelial barrier to the blood-brain-barrier 85. This method can be exploited to assess neurotoxicity, which is a primary concern for new antiviral drugs and vaccines for SARS-CoV-2.

In addition, the SARS-CoV-2 virus itself could hold a neurotropic potential. New observations on the loss of smell and taste by patients later manifesting symptoms of COVID-19, together with evidence showing that coronaviruses such as SARS-CoV and MERS-CoV could target the central nervous system, open up a new window in this regard 86. In fact, a long-term bioreactor co-culture of olfactory neurons and ensheathing glial cells in 3D, could not only confirm the expression of ACE2 in human olfactory neurons, thus their role as a potential entry site for the SARS-CoV-2 virus, but also could help to clarify the potential mechanism of virus propagation to the brain through neural cell populations, *i.e*. from the olfactory neurons to their ensheathing glial cells. Such a model could verify whether, in SARS-CoV-2 infection, the defense mechanism provided by olfactory ensheathing cells is present, and whether it is maintained during aging. Such an experiment could add crucial insights into unveiling the possible contribution of neurological tissue damage to the morbidity and mortality caused by COVID-19 11, also suggesting new prevention measures, such as the vaporization of nasal disinfectants in the elderly population.

### Intravital imaging models *in vivo* for drug testing

Immunization addresses the response of innate and adaptive immune cells to a pathogen, which is a very complex and slowly-developing phenomenon commonly studied *in vivo*. In advanced study models of the immune response to influenza viruses, the experimental animals were directly accessed with a microscope for intravital real time optical inspection of organs including the nasal cavity 87, trachea 88, and lungs 89-91.

Table 6 shows some examples of the intravital imaging models published in the field of immunization. The most elegant procedures generally consist in surgically creating a direct percutaneous access for the microscope objective, often using metallic holders, in transgenic mice with fluorescent CD4^+^ T cells and/or T cell receptors, and in visualizing their interactions with inoculated recombinant vaccine viruses also expressing fluorescent proteins 87-90,92-96. Compared to conventional post-mortem histopathology, several key advantages of intravital imaging techniques can be highlighted in speeding-up the preclinical evaluation of vaccines and novel antiviral therapies.

#### 1) Dynamics of infection progression and the effects of vaccines and drugs

These can be quantified in real time with a resolution at the cellular scale. For example, intravital imaging studies in lungs quantified in time the recruitment of CD4^+^ T cells from blood vessels in response to the influenza A virus 89-90 and, most recently, the recruitment of neutrophils in response to the PR8-H1N1 and H5N5 influenza viruses 91. Multiphoton intravital microscopy in abdominal lymph nodes was used to quantify a partial reduction in the motility of HIV-infected T cells as well as their tethering to and fusion with CD4^+^ immune cells 92-93. Intravital imaging within vessels of colon adenocarcinoma tumors was used to quantify the timely evolution of several oncolytic viruses binding to intravascular leukocytes and endothelial cells 94-95.

#### 2) Sensitivity and robustness of intravital imaging to assess immunization

These parameters are greater than in *in vitro* assays. For example, in an immunization study, using a glycoprotein of the respiratory syncytial virus, associated with an antiviral treatment with the specific monoclonal antibody, live imaging was used to visualize and capture virus-infected cells in the nasal cavity and in the lungs of experimental mice with a higher sensitivity compared to the *in vitro* plaque assay 87. Intravital imaging of live mice was sufficiently sensitive to quantify vaccinia viruses in ear blood vessels and labial mucosa 96, and single oncolytic virus particles interacting with individual leukocytes and endothelial cells within blood, tumors, and visceral organs 94-95.

Intravital imaging is thus emerging as a key tool to observe the spatial-temporal dynamics of very complex phenomena, even as complex as embryonic development, at a cellular resolution 97.

In the field of immunology, however, intravital imaging models have significant limitations. Temporary “suction” window chambers are highly invasive and always require animal euthanization at the end of each observation, preventing repeated observations at subsequent time points. In contrast, implantable windows allow for long-term repeated observations in the same animal. However, they are currently too invasive and affected by chronic inflammation at the surgical site, thus interfering with the desired measurements 88. In addition, implanted imaging windows in general do not retain lymphatic circulation, thus preventing the recruitment of cells from the bone marrow or lymphatic system 89-90.

**Figure 4:**
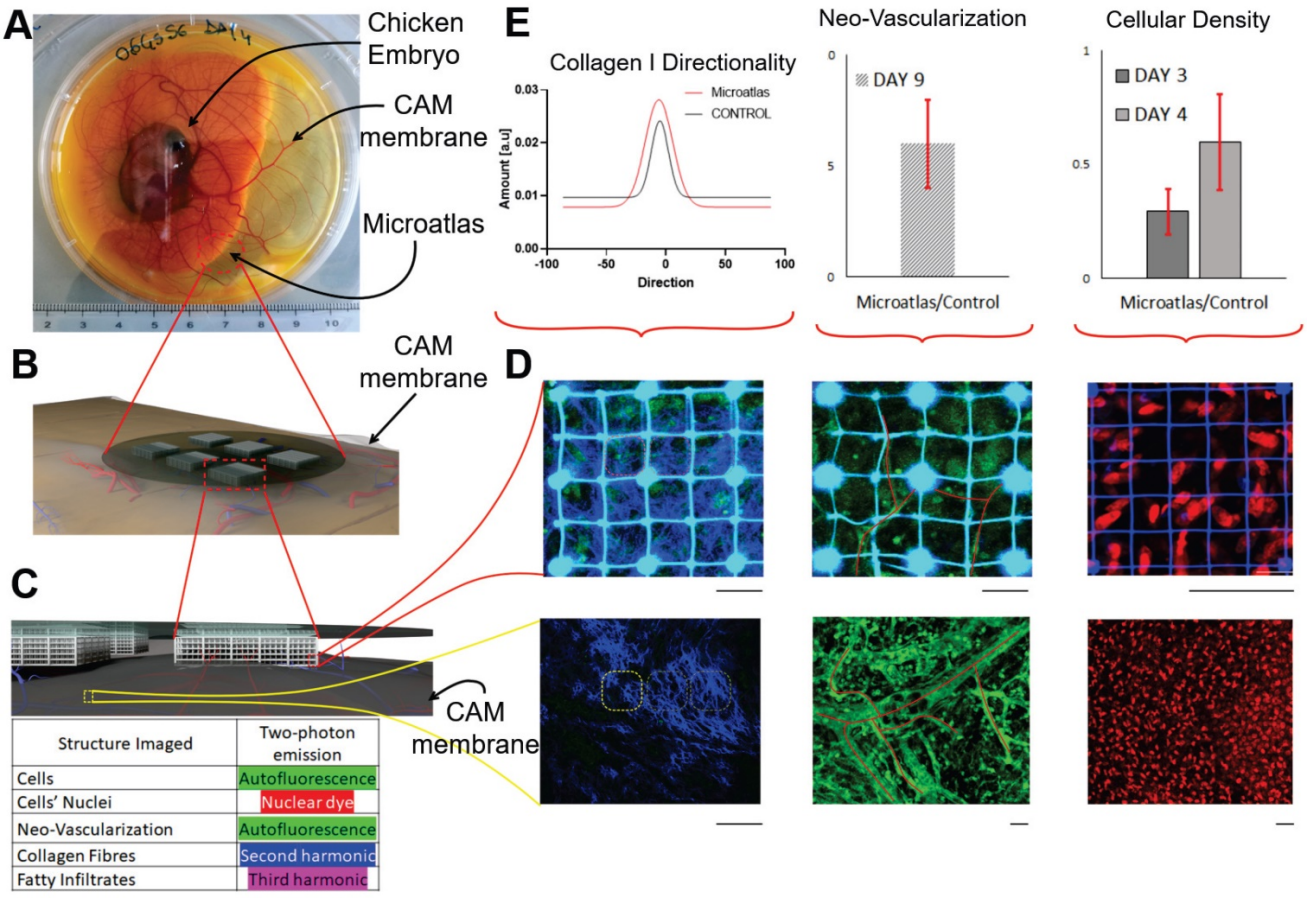
** Miniaturized “Microatlas” imaging window for intravital microscopy. A frontier imaging window device used for mini invasive quantitative analyses of living organisms, without any persistent percutaneous access. The device incorporates micro-scaffolds micro-fabricated by two-photon polymerization. The device was developed for animal examinations with optical fluorescence microscopy techniques. A)** The device was validated by studying the reaction of the foreign body of the implant, and the animal model used was the chicken embryo. The widely employed Chorioallantoic Membrane (CAM) assay was used to quantify and characterize the amount of reaction occurring inside the imaging device. Here an image of a living embryo on the 8^th^ day of incubation: the device was implanted in the membrane (red dot-circle). **B)** Rendering representation of the implant set-up. The device lies on the membrane. No conditioning factors were administered. **C)** Zoomed rendered detail of the tissue infiltration inside the micro-scaffold device. The mechanical conditioning, alone, guides the tissue regeneration in situ, allowing a fast neovascularization in the structure porosity. **D)** Example of possible fluorescence acquisitions that quantify the foreign reaction. In the upper part is the window imaging, with respect to the control, which is reported into the lower part. Second Harmonic Generation (left), tissue autofluorescence (middle) and nuclear dyes (right) were used for the examination in real-time. **E)** Graphical quantitative representation of the reaction occurring in terms of collagen fibers orientation, differential neo-vascularization rate and differential cellular density in subsequent incremental time-points. Scale bars: 50 µm.

To overcome these limitations, we developed an implantable miniaturized intravital imaging device called Microatlas (Figure 4). This minimally invasive device, which is 5 mm in diameter and 1 mm in thickness, is implantable in chicken embryos or subcutaneously in mice to quantify *in vivo* aspects of the immune response to biomaterials and drugs 98. Microatlas integrates a micro-fabricated fluorescent 3D scaffold that guides and hosts the intravital regeneration of vascularized tissue. In addition, this scaffold enables the repositioning of the observation field of view of a two-photon microscope, which is necessary for repeated and quantitative intravital measurements of immune cell recruitment, neo-angiogenesis and fibrotic reaction, on the same animal at different time-points. The fluorescent grid is currently designed to provide beacons to correct aberrations derived from deep optical sectioning of the 3D micro-scaffold repopulated by cells during *ex ovo* experiments.

Expression of the human ACE2 receptor can be engineered in transgenic mice. In contrast with histopathology techniques, imaging through a miniaturized intravital window can be used at incremental time points within the same mouse, in vital conditions to study the localization and distribution, pharmacokinetics, and the pharmacodynamics of antiviral therapies and vaccines. While seeking solutions for COVID-19, it could significantly speed-up the preclinical testing of experimental antiviral drugs and vaccines, while eliminating the need for animal sacrifice at the various time points, thus reducing by up to 90% the number of animals required by the regulation for *ex vivo* assessments.

Intravital imaging could be very effective in this model also to test therapeutic agents based on an engineered messenger RNA (mRNA) that directs cells to make a protein which could work as a drug or vaccine for SARS-CoV-2. Human safety trials have already begun for mRNA-based vaccines against two flu strains and the zika virus. In each case, the mRNA encodes for viral proteins that infected cells would physiologically present to activate the immune system against infections. To succeed, the engineered mRNA must hide from dendritic cells, enter the cells, avoid degradation, and be translated efficiently. The efficiency off all these stages could be monitored and measured by the intravital imaging of fluorescently-labeled mRNA.

## Conclusions

In this review, we have discussed various cutting-edge bioengineering tools already available in the fields of virology and pharmacology, which are based on i) 3D co-cultures that are highly predictive of *in vivo* mechanisms and, ii) high-resolution intravital microscopy. We have also discussed the potential strategies aimed at repurposing these tools in order to significantly accelerate the development of therapeutics and vaccines against COVID-19.

Although several academic institutions have already adopted these improved tools for research into basic immune biology and drug discovery, pharmaceutical companies still use conventional cell lines and standardized animal models for confirmatory studies, as requested by the standards for the passage of new drugs through the preclinical steps of regulatory approval, but despite the limited predictive capability of these models.

The particular urgency to find clinical solutions for the SARS-CoV-2 pandemic makes it even more evident that an adequate validation of these new tools could lead to an update of the current preclinical testing standards, with the new ones based on the concepts of 3D culture, perfused culture and intravital imaging presented here.

In virology and vaccinology, as well as in many other pharmaceutical sectors, the new standards suggested in our review would improve the efficiency and effectiveness of the whole drug development process, with the added benefit of drastically reducing, refining and widely replacing animal testing.

## Supplementary Material

Supplementary figures and tables.

## Figures and Tables

**Table 1:** *In silico* molecular mechanics modeling of drug interaction with SARS-CoV-2

Refs.	Therapeutic agent	Target (Viral subunit)	Suggested drug based on the predicted binding energy
101	Commercially available antiviral drugs.	Mpro, RdRp, helicase, 3'-to-5' exonuclease, endoRNAse 2'-O-ribose methyltransferase.	Atazanavir against all the virus endonucleases.
20	Sofosbuvin and ribavirin, remdesvir, IDX-184 guanosine triphosphate, uracil triphoshate, cinnamaldehyde, thymoquinone.	RdRp.	Sofosbuvir and ribavirin, remdesivir and IDX-184 were shown to tightly bind to the target.
21	7173 purchasable drugs.	Mpro.	Ledipasvir or velpatasvir (minimal side effects) + Epclusa® (velpatasvir/sofosbuvir) and Harvoni® (ledipasvir/sofosbuvir) with inhibitory actions on two viral proteases.
13	Drug repurposing (ZINC database + database of traditional Chinese medicine and natural products).	All the main proteins encoded by SARS-CoV-2: 3-chymotrypsin-like protease (3CLpro), Spike, RdRp and PLpro.	Remdesivir (GS-5734) inhibitor of RdRp. Its efficacy was verified *in vitro*. It is also hypothesized to inhibit Nsp3b (score=-36.5), RdRp (mfScores=-112.8), E-channel (mfScore=-125.1), and TMPRSS2 (score=-36.23, mfScores=-109.4).
102	FDA-approved drugs against viral protease + in-house database of natural and drug-like compounds of synthetic origin.	Mpro	FDA-approved drugs: remdesivir, saquinavir, darunavir + two natural compounds: flavone and coumarin derivatives.
103	Designed inhibitor.	ACE2-Spike interface.	Designed inhibitors consisting of α1+α2 helixes.
104	Designed inhibitor.	Mpro.	Designed drug that needs to be optimized and tested in vitro and vivo.
22	Zinc15 - Approved drugs in major jurisdictions, including the FDA, *i.e.* DrugBank approved.	Spike protein + Mpro.	Modeled the action of many approved drugs: zanamivir, indinavir, saquinavir, remdesivir (against Mpro)+flavin adenine dinucleotide (FAD), adeflavin (against both spike and Mpro), and coenzyme A (against spike protein).
23	Specific drug.	Spike via homology modeling (template PDB: 2GHV)+spike-ACE2 interaction (template: PDB:2AjF).	Cobicistat, carfilzomib and ombitasvir.
24	From a specific drug to its possible target.	20 virus protein + 22 human proteins.	Development of molecular docking based webserver, namely D3Targets-2019-nCoV for target prediction (in vitro/in vivo studies), to identify: (1) the potential target proteins and their different conformations were included; (2) all the potential ligand-binding sites with volume larger than 200 A°^3; (3) correlations among some conformations or binding sites was annotated; (4) easily to be updated, and freely accessible.
25	Medicinal plant library (32, 297 potential anti-viral phytochemicals/traditional Chinese medicinal compounds).	Mpro.	5,7,3',4'-tetrahydroxy-2'-(3,3-dimethylallyl) isoflavone.

**Table 2:** *In silico* prediction of the pathogenic mechanisms of SARS-CoV-2

Refs.	Viral/cellular subunit	Modelling method	Primary results
18	Receptor ACE2 + SARS-CoV-2 spike.	1) Structure alignment between SARS-CoV-2 and SARS-COV; 2) SARS-CoV-2 + ACE2 complex modeling via single point mutation from solved SARS-CoV +ACE2; 3) SARS-CoV-2 and SARS-CoV interaction with ACE2 hot spots (residues mainly involved in RBD binding) comparison; 4) comparison with animals ACE2.	The two spike proteins are quite similar and therefore the authors analyzed in detail the hot spots revealed for SARS-CoV; 2) two virus-binding hot spots identified: ACE2 Hot spot 31(salt bridge Lys31-Glu35) and Hot spot 353 (salt bridge Lys353-Asp38); 3) (a) SARS-CoV-2 Q493 (vs SARS-CoV N479) was compatible but had less of an affinity with hotspot 3, (b) SARS-CoV-2 Q501 (vs SARS-CoV T487) had less affinity with hotspot 353, but was still compatible, (c) SARS-CoV-2 L455 (vs SARS-CoV Y442) preferred interaction with Hotspot 31, (d) SARS-CoV-2 F586 (vs SARS-CoV L472) enhances interaction with Hotspot 31 (e) SARS-CoV-2 S494 (vs SARS-CoV D480) made the interaction weaker but compatible with the hotspot 353; 4) mouse or rat ACE2 contained a hotspot at the 353 position, which did not fit into the RBD of SARS-CoV-2 which instead recognized ACE2 from pigs, ferrets, cats, orangutans, monkeys, and humans.
19	Receptor ACE2 + SARS-CoV-2 spike.	1) Genomic analysis; 2) homology modeling for SARS-CoV-2 spike; 3) structural superimposition in SARS-CoV+ACE2 model; 4) binding free energy evaluation.	1) Low homology in RBD domain between SARS-CoV-2 and SARS-CoV, but high similarity (similar polarity); 2) From free energy binding the authors observed that even though it was weaker than SARS-CoV (-78.6 kcal mol-1), SARS-CoV-2 spike still showed an high affinity with ACE2 (-50.6 kcal mol-1); 3) Differences in aa sequences did not alter the protein conformation and maintained similar Van der Waals and electrostatic properties.
17	receptor CD26 + SARS-CoV-2 Spike.	1) Homology modeling for SARS-CoV-2 Spike (S1+S2) using the SARS-CoV as template (PDB: 6ACD) to obtain inactive configuration and using SARS-CoV+ACE2 complex structure as a template (PDB:6ACG) to obtain the active structure; 2) analysis of glycosylation sites via the server; 3) comparison (SARS-CoV-2 vs SARS-CoV) of the glycosylation shield structure obtained by server; 4) docking between the modeled trimeric spike and human CD26 (PDB: 4QZV).	1) SARS-CoV-2 Spike tetramer configuration in both active and inactive state; 2) Based on the Spike+ACE2 model the authors identified the 3C-like proteinase cleavage site + glycosylation sites; 3) from SARS-CoV and SARS-CoV-2 comparison: SARS-CoV-2 showed several unique glycolysation sites in addition to the conserved from SARS-CoV. This suggested a different shielding or camouflage pattern; 4) SARS-CoV-2 spike showed a large interaction surface with CD26 with many unique residues (different in SARS-CoV) involved in the interaction. SARS-CoV-2, compared to previous SARS-CoV viruses, interacts in a different way with the human receptors.
28	ORF1ab (Nsp2 and Nsp3).	Provides information on how quickly the virus could potentially increase its genetic variability, with implications for contagiousness and drugs via comparison of ORF1ab SARS structure: 1) The ORF1ab of 15 SARS-CoV-2 sequence alignment with five sequences of SARS virus and five sequences from Bat SARS‐like virus; 2) selective pressure analysis; 3) homology modeling for NSp2 Nsp3; 4) trans-membrane analysis.	Potential sites under positive selective pressure were found (501); transmembrane helices were predicted. At the residue 501, Bat SARS‐like coronavirus showed an apolar amino acid (threonine), while SARS and SARS-CoV-2 showed a polar amino acid, glycine and glutamine, respectively. Hypothesis: polarity, and potential to form H‐bonds may confer higher stability to the protein. 723 aa. SARS-CoV-2 sequence showed a Serine replacing for Glycine in Bat SARSlike and SARS coronaviruses. Hypothesis: substitution increases the local stiffness of the polypeptide chain due to a steric effect and to the ability of Serine side chain to form H‐bonds. At 1010 aa, SARS-CoV-2 has Prolin, the Bat SARS‐like coronavirus and SARS virus showed a polar and an apolar a. Hypothesis: steric bulge and stiffness of the Proline may cause local conformation perturbation compared with the proteins of the other two viruses.
27	Spike-ACE2.	Prediction of the interaction ACE2 RBD-spike. Evolutionary analysis and search for the possible virus reservoirs. Comparisons of the spike sequences between SARS‐CoV‐2 with SARS‐CoV, Bat SARS‐like CoV, and other coronaviruses. Analysis of the ACE2 structures and binding motif alignment:1) Alignment of spike protein sequences from different sources. Full-length and RBD sequences of spike protein from SARS‐CoV, bat, or pangolin SARS-like CoV and SARS‐CoV‐2 were aligned; 2) comparison of ACE2 enzyme among different species; 4) prediction of spike protein model and spike‐ACE2 binding model.	Bat SARS‐like CoV RaTG13, is an inner joint neighbor of SARS‐CoV‐2 (6.2% overall genome sequence identity); the full‐length sequence of S pangolin SARS‐like CoV and SARS‐CoV‐2 seem to be a little different but SARS‐CoV‐2 RBD sequence (329 to 521) like CoV, had a higher than 89% similarity with bat SARS‐like CoV; pangolin SARS‐like CoV has a higher probability to cross host barriers and infect humans; from infection-involved aa comparison, they identified pangolins, turtles as possible intermediate hosts.
26	ORF1ab.	1) The authors showed a typical workflow for homology modeling applied to the SARS-CoV-2 ORF1ab proteins; 2) aa sequence comparison. They performed sequence alignment investigations on 10 primary sequences of SARS-CoV-2 via BLAST, SWISS‐MODEL, and Clustal Omega.	1) According to BLAST analysis, the sequence identity of ORF1ab protein between SARS-CoV-2 and SARS‐CoV was over 90% with the query cover of about 100%; 2) the authors showed a typical workflow for homology modeling applied to the SARS-CoV-2 ORF1ab proteins.
29	Spike + ACE2 receptor.	This study explores the binding of the proteins encoded by different human ACE2 allelic variants with SARS‐CoV‐2 spike protein. 1) They selected the coding variants of ACE2 and predicted in silico the possible allelic variants; 2) they modelled the 17 obtained coding variants of ACE2 via homology modeling with native structure and superimposed the structures for comparison; 3) They superimposed the obtained models over the native ACE2‐spike protein complex; 4) inter‐residual interaction maps.	1) The authors selected the allelic structures involved in the ACE2-spike interaction (17 variants); 2) The protein architecture of ACE2 allelic variants was similar to the wild type but the spatial orientation of substituting residues varied notably; 3) ACE2 variants bind with spike protein identical to the native complex + intermolecular contacts between SARS‐CoV‐2 spike protein and ACE2 variants are comparable. Among the differences, rs73635825 (S19P) and rs143936283 (E329G) showed a low binding affinity, which may confer resistance against the virus infection.

**Table 3:** *In vitro* 3D cell models of viral infection

Refs.	Pathogen	Cell type/s	Cell culture configuration
30	SARS-CoV-2.	Primary human epithelial cells from airway epithelium.	Epithelial cells were isolated and expanded in transwells, then an air-liquid-interface (ALI) model was provided with a pseudo-stratified and fully-differentiated system, showing basal, ciliated and goblet cells which secreted mucus and proteins.
33	SARS-CoV-2.	Primary human cholangiocytes from primary bile ducts.	Liver ductal organoids (LDO) embedded in Matrigel.
46	MERS-CoV.	Primary human intestinal epithelial cells, LGR5^+^ intestinal adult stem cells and Caco2 cells.	A) 2D epithelial intestinal cell (2D/EICs) differentiation for 7 days. B) Human intestinal tissue (HTI) excised and maintained in transwells coated with Matrigel. C) Intestinal Organoid from LGR5+ cells embedded in Matrigel. D) Caco-2 cells seeded on transwells and polarized (2 weeks).
105	Zika virus.	Primary human testicular cells: spermatogonial stem cells (SSC), Sertoli cells (SC), Leydig cells (LC) and peritubular cells.	Human testicular organoid (HTO): SSC, SC, LC and peritubular cells centrifugated and seeded in ultra-low attachment plate maintained with medium + testis ECM after 48 h self-assembled in spheroids.
35	Respiratory syncytial virus (RSV).	Human iPSCs from fetal tissue.	Lung bud organoid (LBO) from hPSCs embedded in Matrigel and induced to anterior foregut endoderm differentiation (CD184^+^ +CXCR4/c-KiT) and airway branch formation.
36	Respiratory syncytial virus (RSV).	Primary epithelial cells isolated from non-small cell lung cancer.	Cells were embedded in basement membrane extract (BME) and grown as adult human epithelial airway organoids (pseudostratified airway epithelium with basal, secretory, multi-ciliated cells).
37	Avian influenza (IAV) H1N1/H3N2.	Primary human small airway epithelial cells (SAEpCs).	HSAEpCs were loaded onto a 3D chitosan-collagen scaffold and exposed to air-liquid interface (ALI) on the upper part of transwell, alternatively the cells were totally covered with media. 2D culture conditions were performed by seeding cells directly on the upper part of a transwell plate replicating the same ALI and immerged conditions as the 3D one.
38	Avian influenza (IAV) (H1N1 - H7N9/Ah - H5N1 - H7N2 -H7N9/ah).	Human lung resident adult stem cells (ASC).	Airway organoids and proximally differentiated airway organoids (AO) made by ASCs embedded in Matrigel and grown in transwells for 16 days.
39	Parainfluenza virus-3 (HPIV3) + measles virus (MeV).	A) Human airway epithelium (HAE) system: normal human derived trachea/bronchial epithelial cells; B) Organoid system: embryonic stem cell line 2 (RUES2) + feeder layer mouse embryonic fibroblasts.	A) HAE system: epithelial cells cultured to form a highly differentiated pseudostratified mucociliary epithelium; B) lung bud organoids grown to the last-second trimester of lung development (LBO): endoderm (c-KiT, CXCR4 positive)+anterior endoderm foregut formation + ventralization inductions followed by branching airways formation after Matrigel embedding.
45	Measles virus (MeV).	Human primary fibroblast (FBCs)+H358 lung adenocarcinoma epithelial cell line+dendritic cells (DCs) human primary monocytes.	Decellularized porcine small intestinal submucosa (SIS) seeded on the apical side with FBCs and H358-eFLUOR670 cells, DCs-GFP^+^ and DCs-GFP- added in suspension on the basolateral side.
48	HIV-1 virus.	Primary human CD4^+^ T-lymphocytes.	Cells were loaded in custom-made collagen type I gels derived from rat tail and bovine.
40	Andes-hantavirus (ANDV).	Human lung fibroblast MRC-5+h-bronchial epithelial cells 16HBE14o-.	Layered construct in transwell system: bottom bovine collagen-I, MRC-5 + b-coll-I, top 16HBE14o- guarantee air-liquid interface (ALI). ANDV inoculated from the apical side of the construct.
41	Human rhinovirus C (HRV-C) and human bocavirus (HBoV).	Human primary epithelial cells (HAE).	2D culture systems provided via HAE seeding in monolayers. 3D model obtained seeding HAEs in 4 different ways on transwells coated with Matrigel. 3D possible configurations of HAEs were: ON, WITHIN, ON and WITHIN and UNDER Matrigel.

**Table 4:** *In vitro* 3D cell models of viral infection and evaluation of antiviral therapy effects

Refs.	Pathogen	Therapeutic agent	Cell type/s	Cell culture configuration
31	SARS-CoV-2.	Remdesivir, remdesivir + diltiazem.	A) Vero E6 cell line for monolayer culture, B) nasal and bronchial human primary epithelial cells for pseudostratified epithelial model.	A) Vero E6 cultured in standard static condition, B) cells after isolation cultured in transwell inserts to reach a full differentiation until pseudostratified mucociliary epithelium (human airway epithelium HAE).
32	SARS-CoV-2, SARS-CoV, MERS-CoV.	β-D-N4-hydroxycytidine (NHC, EIDD-1931).	A) 2D model with Calu-3 human lung epithelial cell line; B) human primary tracheobronchial epithelial cells (HAE).	Human airway epithelium cultures (HAEs) were generated by an air-liquid interface for 6 to 8 weeks to form well- differentiated, polarized cultures that resembled in vivo mucociliary epithelium. Primary cells were expanded and plated at a density of 250,000 cells per well on transwell-COL (12mm diameter) supports.
34	SARS-CoV-2.	Human recombinant soluble angiotensin converting enzyme 2 (hrsACE2).	iPSCs and human embryonic stem cells (hESCs). For bidimensional culture: Vero-E6 cells.	Human capillary organoids from iPSCs and kidney organoids form hESCs.
42	H7N9 influenza A virus.	Recombinant human interferons α2b (rhIFN-α2b) and λ1 (rhIFN-λ1).	Well-differentiated pseudostratified human airway epithelium (HAE) cells; controls: A549 adenocarcinomic human alveolar basalepithelial cell line.	All cell types were grown at the air-liquid interface: 2D HAE in monolayer on collagen, 3D HAE in pseudostratified layers on transwell; A549 on transwell.
43	Rhinovirus species (RV-A16, RV-B14 and RV-C15); enteroviruses EV-D68 and influenza A (H1N1 and H3N2).	Rupintrivir, an irreversible inhibitor of human rhinovirus protease, was chosen as a reference drug. Oseltamivir, a neuraminidase inhibitor, approved to treat and prevent influenza infections.	Airway cells were obtained from 14 different patients undergoing surgical polypectomy.	Cells were cultured at the air-liquid interface in MucilAir culture medium in 24-well plates with 6.5-mm transwell inserts.
44	Syncytial virus (RSV).	Entry/fusion inhibitors (GS-580619 and TMC353121); nucleoside viral polymerase inhibitor; two non-nucleoside replication inhibitors (AZ-2721 and PC786).	Fully-differentiated human airway epithelium cells (HuAECs) of bronchial origin from healthy donors.	HuAECs of bronchial origin were seeded in an air-liquid interface cell culture system with MucilAir medium.
49	Rotavirus.	Interferon-alpha (IFN-α) and ribavirin.	For mouse and human organoids: resection on small intestine of mouse and intestinal biopsies or surgically resected intestinal tissues for patients; for 2D culture: Caco2 cell line.	The crypts obtained from the biopsies were suspended in growth factors reduced phenol-red free Matrigel. This was placed in the center of each well of a 24-well plate and was subsequently incubated with a specific culture medium.
47	Hepatitis B (HBV).	Entecavir.	HepG2-NTCP cell line (transfected cell line for HBV infection) + primary human hepatocytes (PHHs).	Human decellularized liver scaffold from healthy and cirrhotic donors (hDCS-hDLCS) seeded with PHHs and with HepG2-NTCP.
50	Herpes Simplex virus 1 (HSV-1).	Antivirals 5BVdU and interferon-alpha (IFN-α).	Human fibroblast derived iPSCs and HFF1S cell lines.	2D neural culture obtained by hiPSCs seeded and cultured with neurobasal medium and brain neurothrophic factor to become neural progenitor cells (NPCs) and mature neurons. 3D brain organoids obtained via hiPSCs differentiation towards NPCs self-assembled neurospheroids in transwell and maturing in a toroidal shape while cultured in low-attachment plates.
51	Herpes simplex virus-1 (HSV-1).	Acyclovir (ACV).	3T3 fibroblasts; for viral stock: Vero cell lines.	In vitro 3D acrylated hyaluronic acid (AHA) hydrogel model encapsulating fibroblasts.
99	Herpes simplex virus type 1 and type 2 (HSV) and varicella-zoster virus (VZV).	Acyclovir, penciclovir, brivudin, foscarnet, and cidofovir.	Primary human keratinocytes isolated from neonatal foreskins.	Organotypic epithelial raft cultures that permit full differentiation of keratinocyte monolayers. The cells were cultures on collagen gels at the air-liquid interface.
100	Cowpox virus.	Gefitinib.	Pooled primary normal human epidermal keratinocytes (NHEK); for viral stock: HEp-2 cells and Vero E6 cells.	For 3D culture, decellularized equine pericardium used as a biological ECM for cell culture of 1×10^5^ NHEK cells. For monolayer control, NHEK were seeded in 24-well cell culture plates.

**Table 5:** *In vitro* cell models of viral infection based on bioreactor-perfused cultures

Refs.	Pathogen	Cell type/s	Cell culture configuration
67-68	SARS-CoV.	Primary human bronchial-tracheal cells (HBTCs) used to establish a human lung tissue-like assembly (TLA) with an overlay of human bronchial epithelial (BEAS-2B) cells. 2D control established with BEAS-2B monoculture.	3D tissue dynamic culture system established using a Rotating Wall Vessel (RWV) bioreactor. Static 2D controls: tissue culture flasks of near confluent BEAS-2B monocultures.
70	Hepatitis B virus (HBV).	Primary Human Hepatocytes (PHH) and co-culture with primary Kupffer cells (KC).	Perfused 3D bioreactor. Medium was recirculated via a pneumatically driven micro-pump, and collagen-coated scaffolds were used for cell adherence. Each plate consisted of 12 individual bioreactors, in which the flow rate was regulated. 3D perfused: PHH seeded in 3D scaffolds. Non-perfused controls: 3D spheroid, static 2D PHH, and self-assembling co-cultures (SACC) of PHH.
68-69	Varicella zoster virus (VZV).	Normal human neural progenitor (NHNP) cells used to establish human neuronal tissue- like assembly (TLA). Gene mutations, infection study and VZV genome maintenance were performed on the TLA and validated with a 2D control of human melanoma (MeWo) cells.	Optimized 3D tissue culture systems created using a Rotating Wall Vessel (RWV) bioreactor. The 3D model was compared to a 2D control of NHNP cells.
71	Adenovirus carrying enhanced green fluorescent protein (AdEGFP).	Mouse embryonic fibroblasts (MEFs) and human foreskin fibroblasts (HFFs) both for microfluidic study and static control.	Microfluidic device composed of a supporting glass slide with a PDMS slab carved to accommodate the cell culture coverslip, a membrane-based vacuum system for the reversible sealing, and microfluidic channels delivering fluids to the cultured cells. The control was performed in static conditions.
106	Enhanced green fluorescent protein (EGFP) plasmid.	3T3 fibroblasts used for 2D control, static and dynamic conditions.	Microfluidic perfused device for dynamic cell culture and virus infection in a micro channel, control was performed in 2D conventional culture, and in the microfluidic device was evaluated in both static and dynamic conditions.

**Table 6:** *In vivo* models of viral infection based on intravital imaging

Refs.	Pathogen	Function monitored	Imaged Cell type/s	Imaging window configuration	Imaged Organ/District
91	Influenza viruses: PR8-H1N1, PR8-H5N1.	Correlated pathological changes in the lungs of live animals at the cellular level (labeled immune-related cells) in response to influenza viruses.	Madin-Darby canine kidney cells for virus propagation.Mice immune-related cells.	Thoracotomy incision and left lung immobilization by vacuum.	Left lung.
89-90	Influenza A virus (IAV).	CD4^+^ T cells responding to IAV in the mice lung.	Mouse transgenic CD4^+^ T cells.	Thoracotomy incision and left lung immobilization by vacuum.	Lung.
88	Influenza virus (PR8- H1N1).	Neutrophil and dendritic cell interactions with influenza virus at the tracheal site.	Neutrophils, dendritic cells.	Tracheal exposure by removing the tissue and muscles.	Trachea.
87	Respiratory Syncytial Virus (RSV) A2-Line19F.	Kinetics of RSV infection in the nasal cavity and in the lungs of live BALB/c mice infected with a recombinant virus strain expressing firefly luciferase gene (RSV A2-Line19FFL). Immunization versus therapy by either vaccination with 5 μg of DS-Cav1, a glycoprotein of the syncytial virus, or treatment with the monoclonal antibody Palivizumab.	Virus expansion: A549, Hep-2.	Whole body live imaging without specific localization of window.	Whole body imaging detected strong bioluminescencesignal in the nasal cavity and in the lungs.
92-93	HIV.	HIV infection on T cell migration.	HEK293T cell line (virus expansion), Human CD4^+^ central memory-like T cells.	Abdominal exposure with permanent imaging device implanted.	Popliteal lymph nodes (popLN).
94-95	Oncolytic Viruses (OV): vesicular stomatitis virus (VSV); maraba virus; reovirus.	Interaction between OV and tumor *in vivo*.	Virus expansion: Vero cells (Vesicular Virus); L929 (Reovirus). Tumor model: CT-26 (murine colon adenocarcinoma).	Dorsal/Abdominal exposure with no permanent imaging device implanted.	Skin/endothelium.
96	Vaccinia virus (VACV).	VACV infection of mice to study both the movement of infected cells and the response of innate and adaptive immune cells into skin and mucosa.	Infected cells.	Craniocaudal exposure: (1) Ear sealing with no external device or wound generation; (2) Lower labial mucosa exposed into a steel stage.	(1) Ear blood vessels; (2) Labial mucosa.

## Abbreviations

3CLpro: 3-chymotrypsin-like protease
ACE2: angiotensin-converting enzyme 2
ACV: acyclovir
ADRP: ADP ribose phosphatase
AHA: acrylated hyaluronic acid
ALI: air-liquid-interface
ANDV: andes-hantavirus
AO: airway organoids
ASC: adult stem cells
BME: basement membrane extract
CAD: computer aided design
CAM: chorioallantoic membrane
CD: cluster differentiation
CFD: computational fluid dynamics
CoV: coronavirus
COVID-19: coronavirus disease 2019
CPU: central processing unit
cryo-EM: cryo-electron microscopy
DC: dendritic cell
E: envelope protein
ECM: extracellular matrix
EGFP: enhanced green fluorescent protein
EIC: epithelial intestinal cell
EM: electron microscopy
EV: enterovirus
FACS: fluorescent-activated cell sorter
FAD: flavin adenine dinucleotide
FBC: human primary fibroblast
FP: fusion peptide
GFAP: glial fibrillary acidic protein
hADSC: human adipose derived stem cell
HAE: human airway epithelium
HBoV: human bocavirus
HBsAg: hepatitis B surface antigen
HBTC: human bronchial-tracheal cell
HBV: hepatitis b virus
hESC: human embryonic stem cell
HFF: human foreskin fibroblasts
HIV: human immunodeficiency virus
HPIV3: parainfluenza virus-3
HR1: heptad repeat 1
HR2: heptad repeat 2
hrsACE2: human recombinant soluble ACE2
HRV-C: human rhinovirus c
HSAEpCs: human primary small airway epithelial cells
HSV: herpes simplex virus
HTI: human intestinal tissue
HTO: human testicular organoid
HuAEC: human airway epithelium cell
IAV: avian Influenza
IC: intracellular domain
ICAM-1: intercellular adhesion molecule 1
IFI44L: interferon-induced protein 44-like
IFN-α: interferon-α
IL-6: interleukin 6
IL-8: Interleukin 8
IL1RN: interleukin 1 receptor antagonist
iPS: Induced pluripotent stem cells
KC: kupffer cells
KRT: keratin
LBO: lung bud organoid
LC: leydig cells
LDO: liver ductal organoids
M: membrane protein
MEF: mouse embryonic fibroblast
MERS: middle east respiratory syndrome
MeV: measles virus
MOAB: miniaturised optically accessible bioreactor
Mpro: main protease
MSC: mesenchymal stem cell
N: nucleocapsid protein
NF-κB: nuclear factor kappa-light-chain-enhancer of activated B cells
NHEK: normal human epidermal keratinocytes
NHNP: normal human neural progenitor
NPC: neural progenitor cell
NSP: non-structural proteins
NTD: N-ter domain
OAS1: 2'-5'-Oligoadenylate Synthetase 1
ORF: open reading frame
OV: oncolytic viruses
PCR: polymerase chain reaction
PDB: protein data bank
PDGFR-α: platelet-derived growth factor receptor α
PDGFR-β: platelet-derived growth factor receptor β
PDMS: polydimethylsiloxane
PHH: primary human hepatocytes
Plpro: papain-like protease
popLN: popliteal lymph nodes
RBD: receptor binding domain
RBM: receptor binding motif
RdRp: RNA-dependent RNA polymerase
rhIFN: recombinant human interferon
RSV: respiratory syncytial virus
RV: rhinovirus
RWV: rotating wall vessel
S: spike protein
SACC: self-assembling co-culture
SARS: severe acute respiratory syndrome
SC: sertoli cells
SD: subdomain
SEM: scanning electron microscopy
SIS: small intestinal submucosa
SSC: spermatogonial stem cell
TLA: tissue-like assembly
TM: transmembrane region
TMPRSS2: transmembrane serine protease 2
TNFα: tumor necrosis factor alpha
VACV: vaccinia virus
VEGF-A: vascular endothelial growth factor A
VSV: vesicular stomatitis virus
VZV: varicella-zoster virus
WSS: wall shear stress
α-SMA: alpha-smooth muscle actin
