# Supplementary material for: Bioengineering tools to speed up the discovery and preclinical testing of vaccines for SARS-CoV-2 and therapeutic agents for COVID-19
Source: Theranostics. 2020 May 27;10(16):7034–52. doi: 10.7150/thno.47406 (PMC7330866; doi:10.7150/thno.47406)

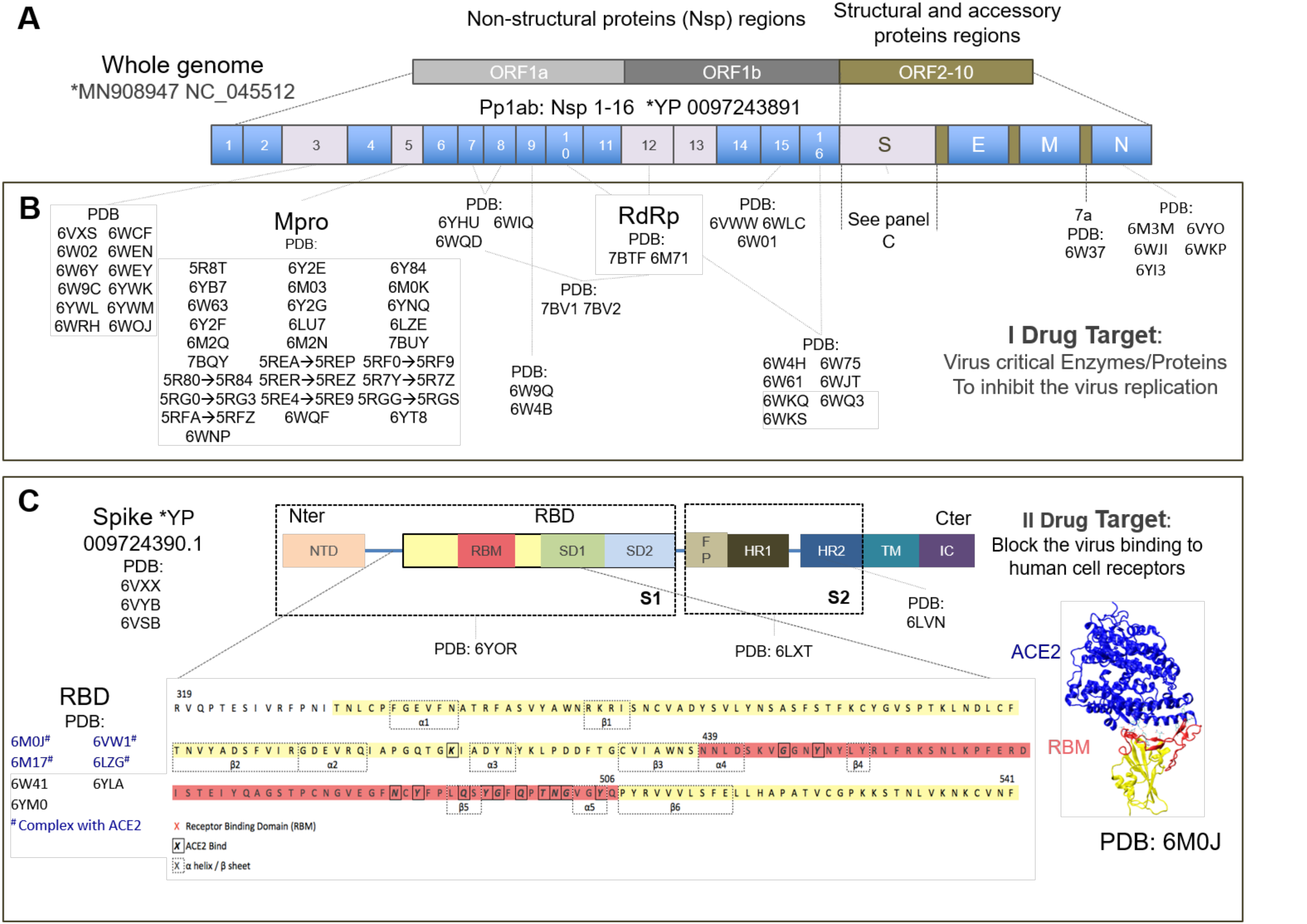
A
Whole genome
*MN908947 NC_045512
Non-structural proteins (Nsp) regions
Structural and accessory proteins regions
ORF1a
ORF1b
ORF2-10
Pp1ab: Nsp 1-16 *YP 0097243891
1 2 3 4 5 6 7 8 9 10 11 12 13 14 15 16 S E M N
B
PDB
6VXS 6WCF
6W02 6WEN
6W6Y 6WEY
6W9C 6YWK
6YWL 6YWM
6WRH 6WOJ
Mpro
PDB:
5R8T 6Y2E 6Y84
6YB7 6M03 6M0K
6W63 6Y2G 6YNQ
6Y2F 6LU7 6LZE
6M2Q 6M2N 7BUY
7BQY 5REA→5REP 5RF0→5RF9
5R80→5R84 5RER→5REZ 5R7Y→5R7Z
5RG0→5RG3 5RE4→5RE9 5RGG→5RGS
5RFA→5RFZ 6WQF 6YT8
6WNP
PDB:
6YHU 6WIQ
6WQD
RdRp
PDB:
7BTF 6M71
PDB:
7BV1 7BV2
PDB:
6W9Q
6W4B
PDB:
6VWW 6WLC
6W01
PDB:
6W4H 6W75
6W61 6WJT
6WKQ 6WQ3
6WKS
See panel C
7a
PDB:
6W37
PDB:
6M3M 6VYO
6WJI 6WKP
6YI3
I Drug Target:
Virus critical Enzymes/Proteins
To inhibit the virus replication
C
Spike *YP 009724390.1
PDB:
6VXX
6VYB
6VSB
Nter
RBD
NTD
RBM
SD1
SD2
S1
F P
HR1
HR2
TM
IC
S2
Cter
PDB: 6YOR
PDB: 6LXT
PDB:
6LVN
II Drug Target:
Block the virus binding to human cell receptors
ACE2
RBM
PDB: 6M0J
RBD
PDB:
6M0J# 6VW1#
6M17# 6LZG#
6W41 6YLA
6YM0
Complex with ACE2
319
R V Q P T E S I V R F P N I T N L C P F G E V F N A T R F A S V Y A W N R K R I S N C V A D Y S V L Y N S A S F S T F K C Y G V S P T K L N D L C F
α1 β1
439
T N V Y A D S F V I R G D E V R Q I A P G Q T G K I A D Y N Y K L P D D F T G C V I A W N S N N L D S K V G G N Y N Y L Y R L F R K S N L K P F E R D
β2 α2 α3 β3 α4 β4
506 541
I S T E I Y Q A G S T P C N G V E G F N C Y F P L Q S Y G F Q P T N G V G Y Q P Y R V V V L S F E L L H A P A T V C G P K K S T N L V K N K C V N F
β5 α5 β6
X Receptor Binding Domain (RBM)
X ACE2 Bind
X α helix / β sheet

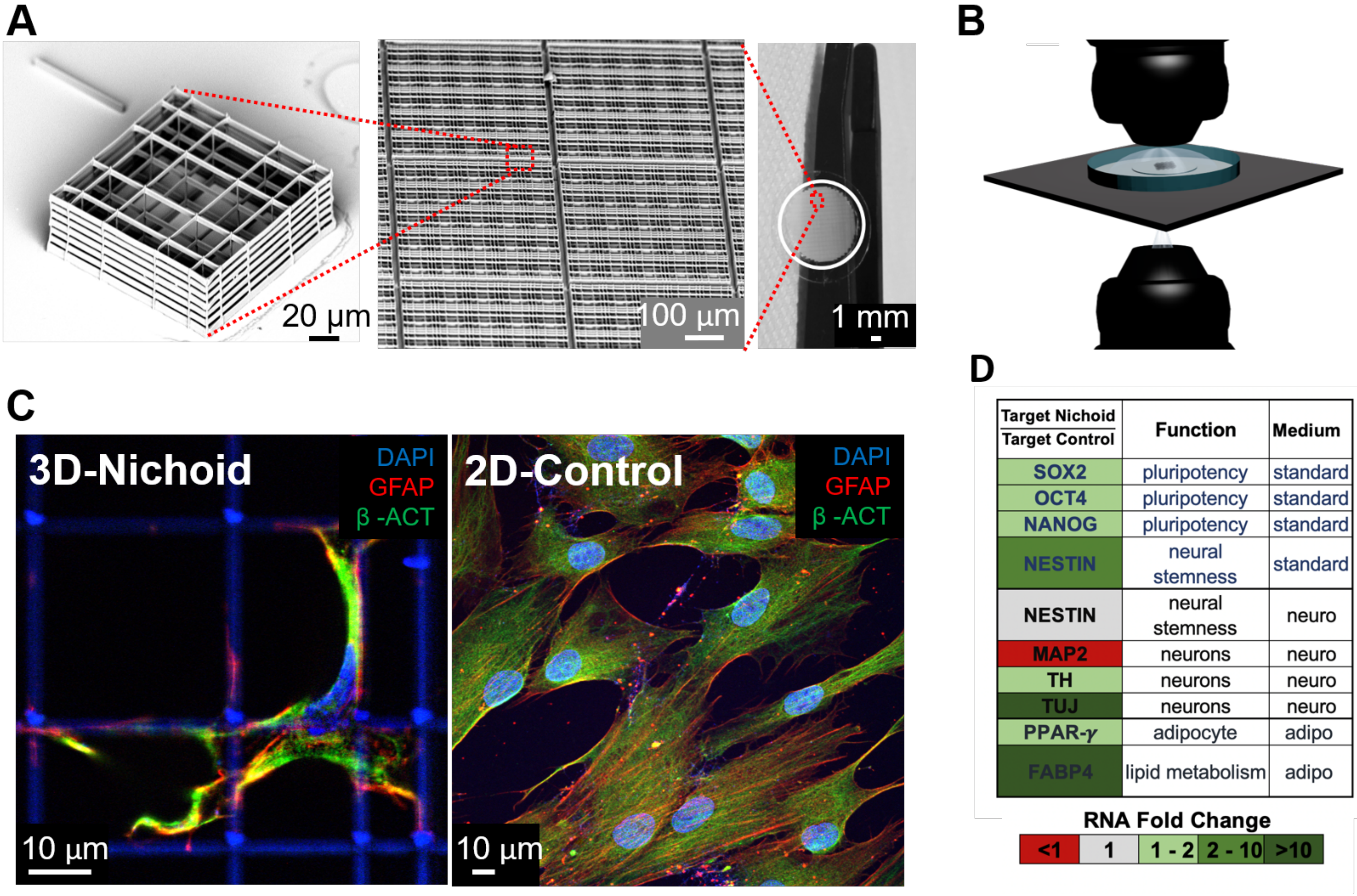
A
20 µm
100 µm
1 mm
B
C
3D-Nichoid
DAPI
GFAP
β -ACT
10 µm
2D-Control
DAPI
GFAP
β -ACT
10 µm
D
Target Nichoid / Target Control
Function
Medium
SOX2 pluripotency standard
OCT4 pluripotency standard
NANOG pluripotency standard
NESTIN neural stemness standard
NESTIN neural stemness neuro
MAP2 neurons neuro
TH neurons neuro
TUJ neurons neuro
PPAR-γ adipocyte adipo
FABP4 lipid metabolism adipo
RNA Fold Change
<1
1
1 - 2
2 - 10
>10

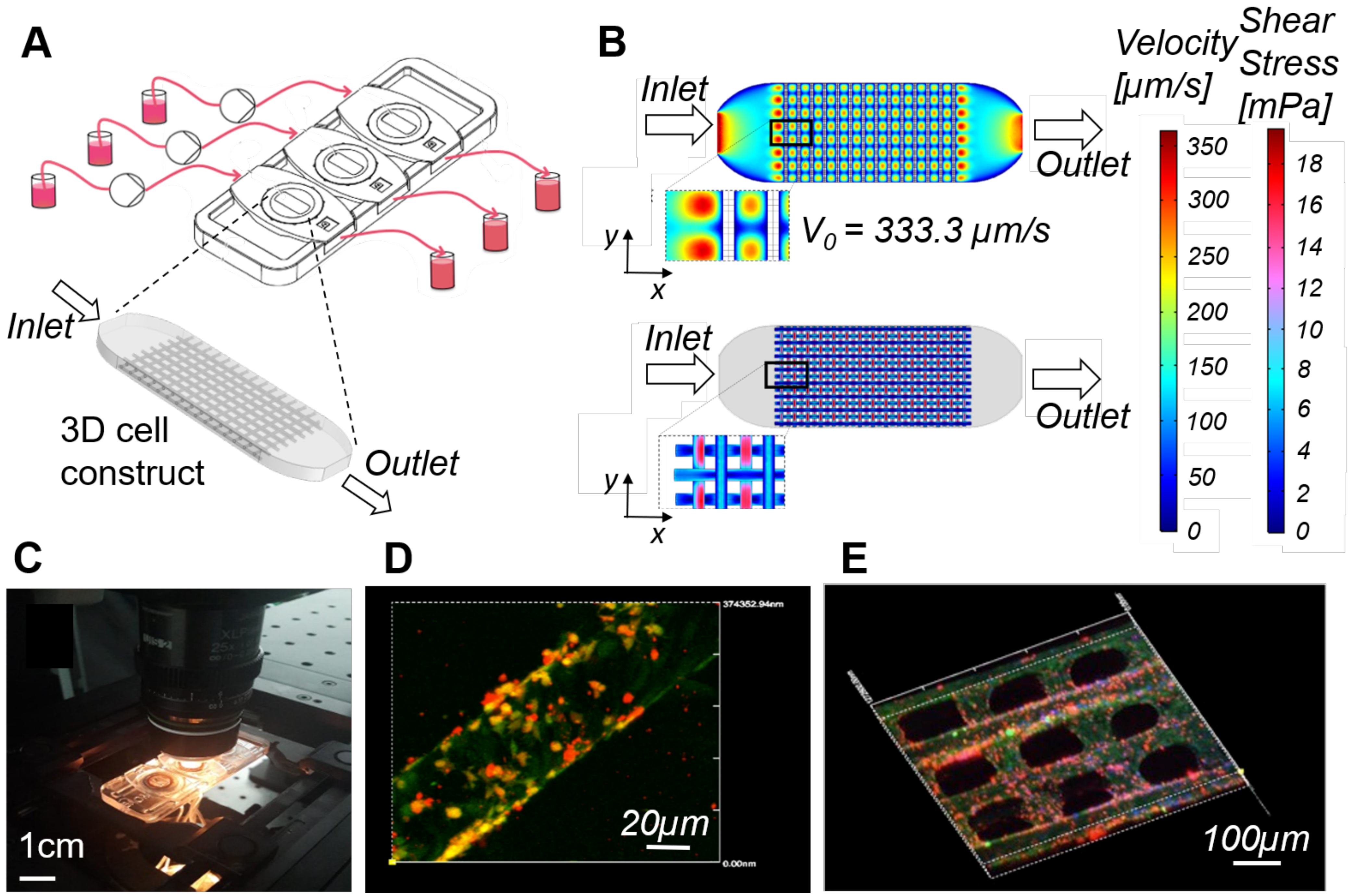
A
Inlet
3D cell construct
Outlet
B
Inlet
Outlet
V_0 = 333.3 μm/s
y
x
Inlet
Outlet
y
x
Velocity [μm/s]
350
300
250
200
150
100
50
0
Shear Stress [mPa]
18
16
14
12
10
8
6
4
2
0
C
1cm
D
374352.94nm
0.00nm
20μm
E
100μm

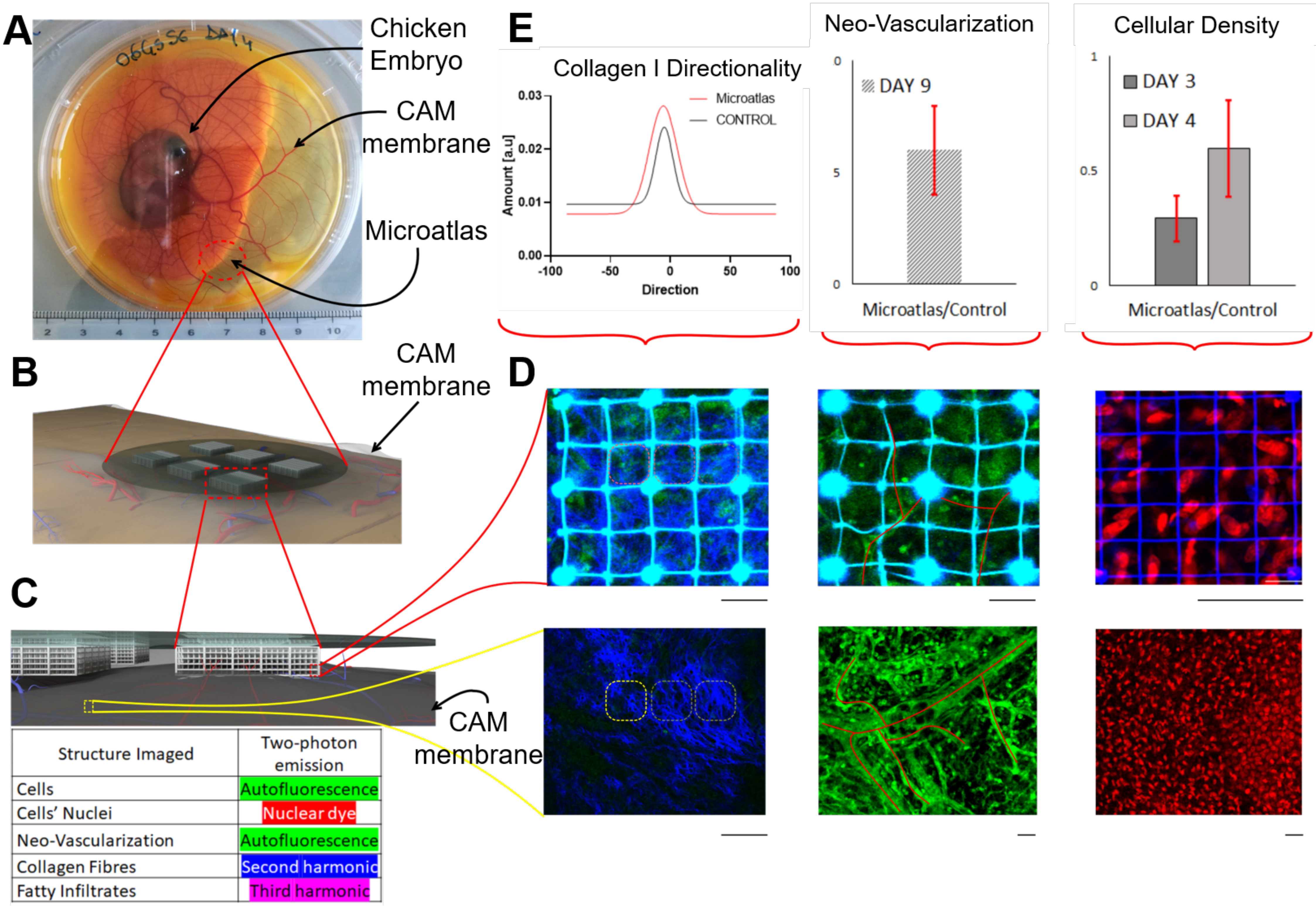
A
Chicken Embryo
CAM membrane
Microatlas
0645 56 DAY 4
B
CAM membrane
C
CAM membrane
Structure Imaged
Two-photon emission
Cells
Autofluorescence
Cells' Nuclei
Nuclear dye
Neo-Vascularization
Autofluorescence
Collagen Fibres
Second harmonic
Fatty Infiltrates
Third harmonic
E
Collagen I Directionality
Microatlas
CONTROL
Amount [a.u]
0.03
0.02
0.01
0.00
-100
-50
0
50
100
Direction
Neo-Vascularization
DAY 9
0
5
0
Microatlas/Control
Cellular Density
DAY 3
DAY 4
1
0.5
0
Microatlas/Control
D

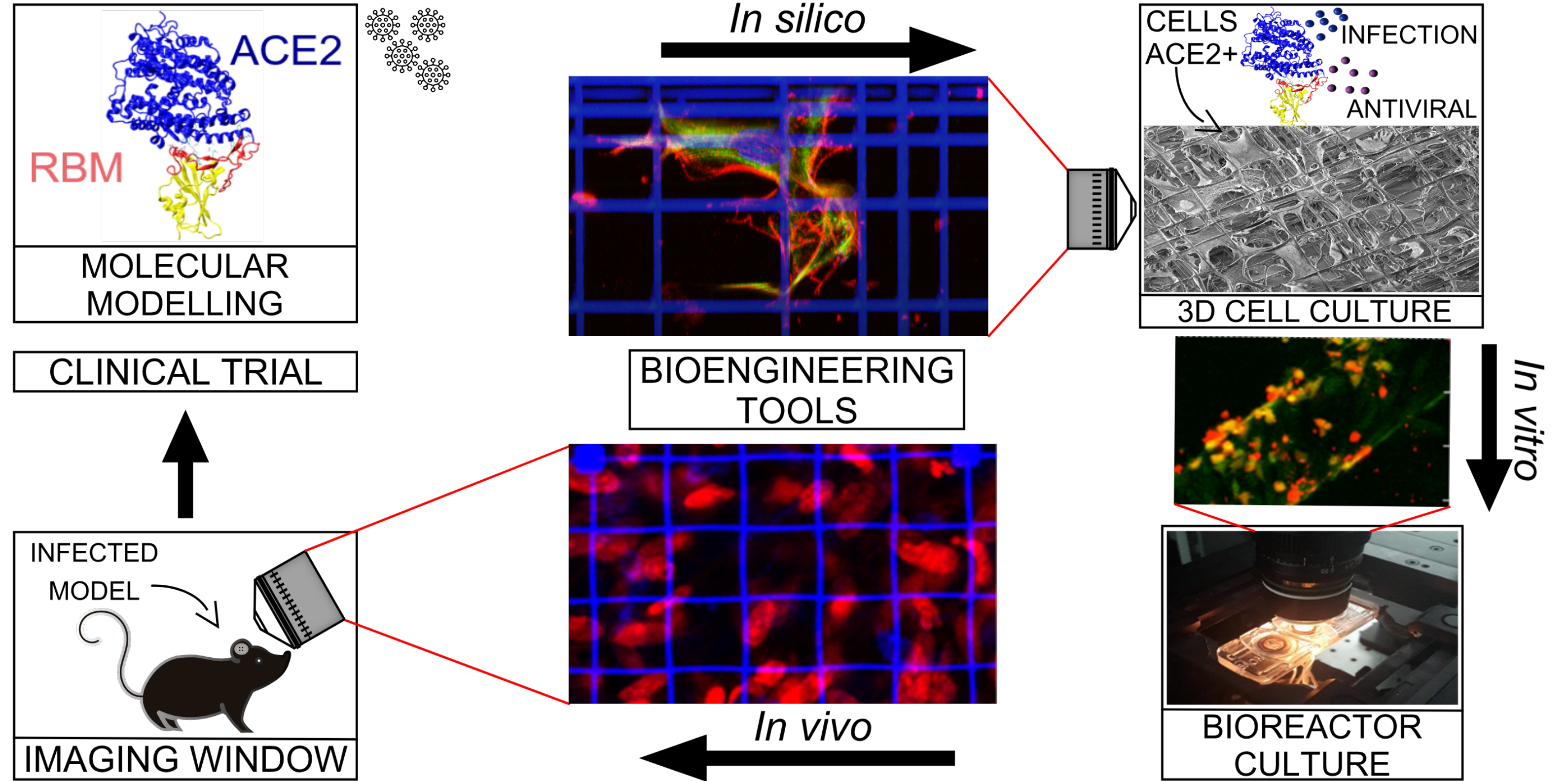
ACE2
RBM
MOLECULAR MODELLING
In silico
CELLS ACE2+
INFECTION
ANTIVIRAL
3D CELL CULTURE
CLINICAL TRIAL
BIOENGINEERING TOOLS
In vitro
INFECTED MODEL
IMAGING WINDOW
In vivo
BIOREACTOR CULTURE

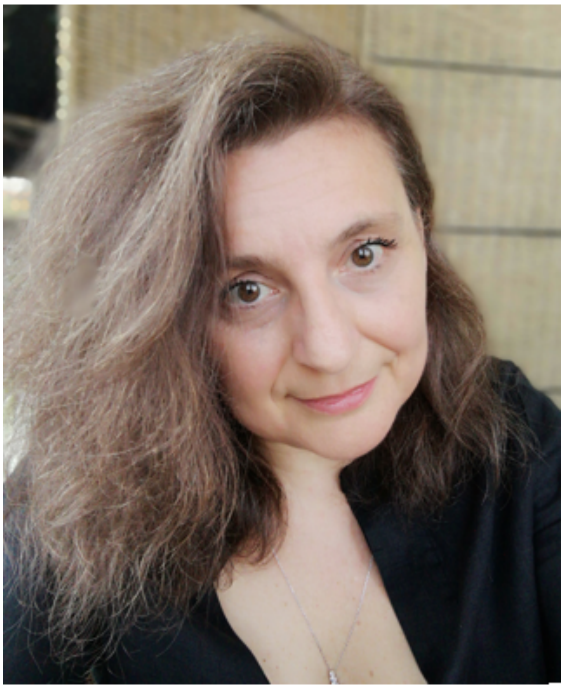

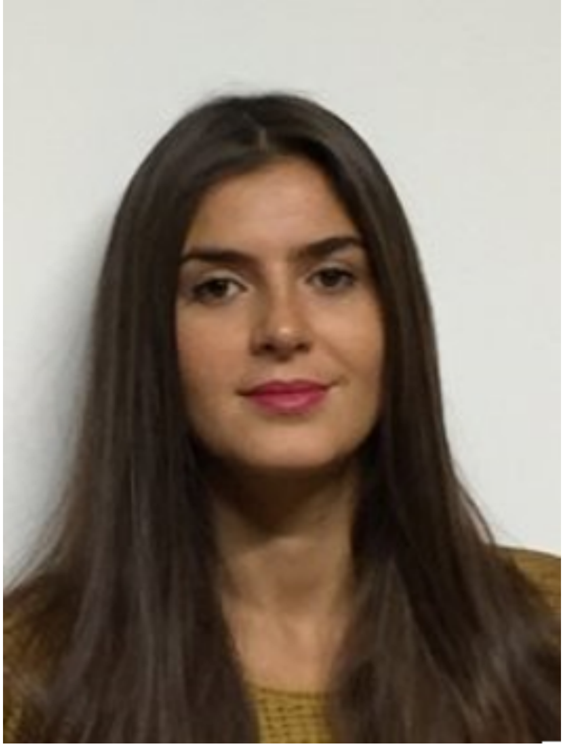

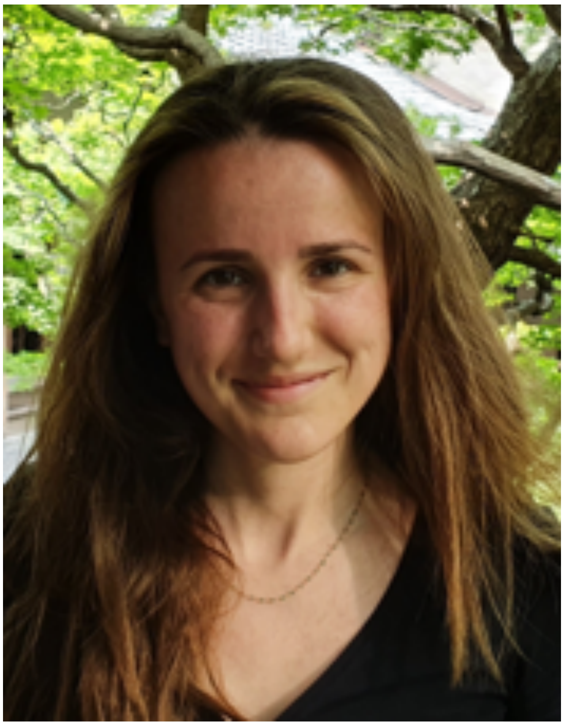

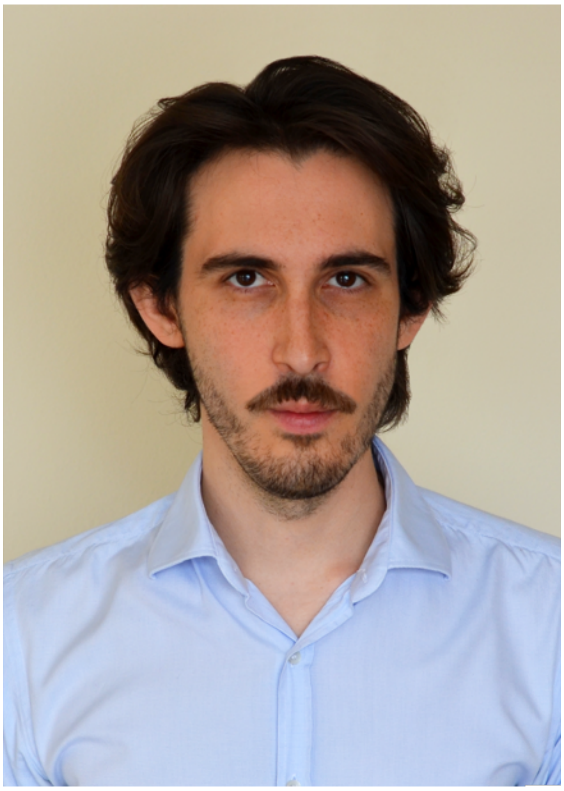

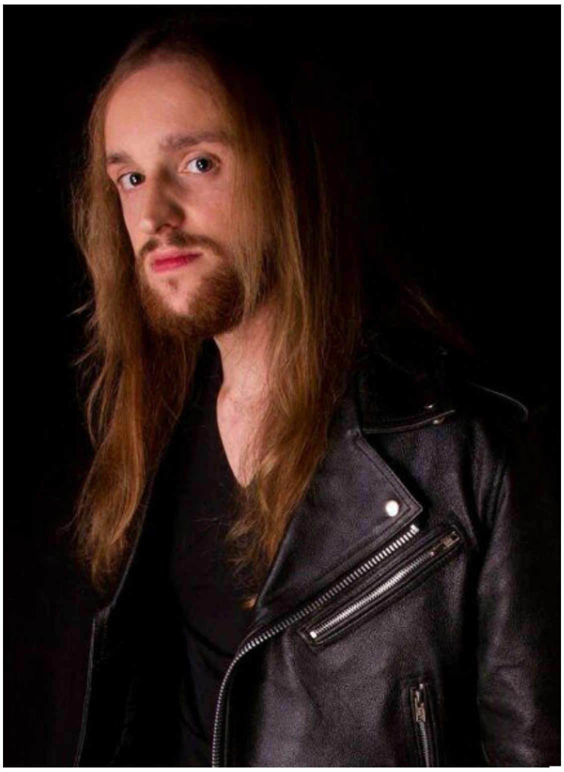

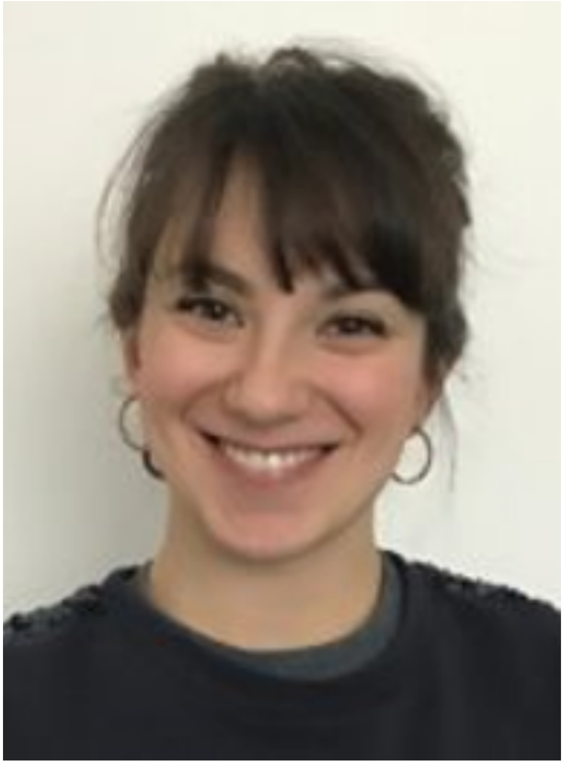

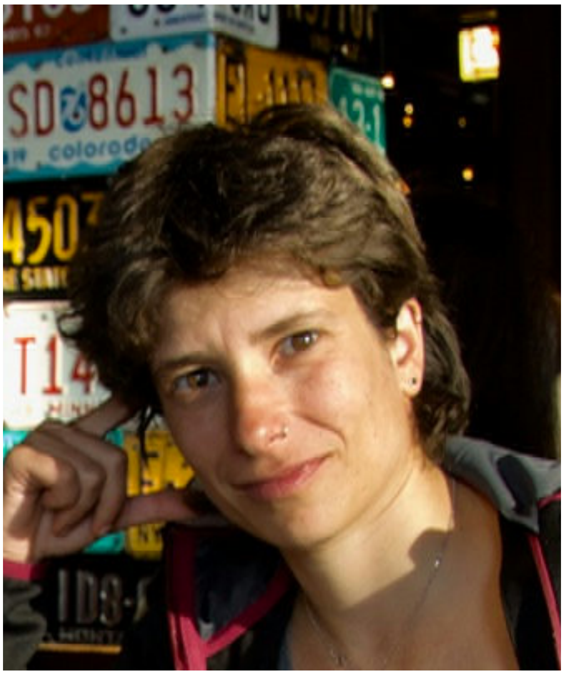
SD 8613
colorado

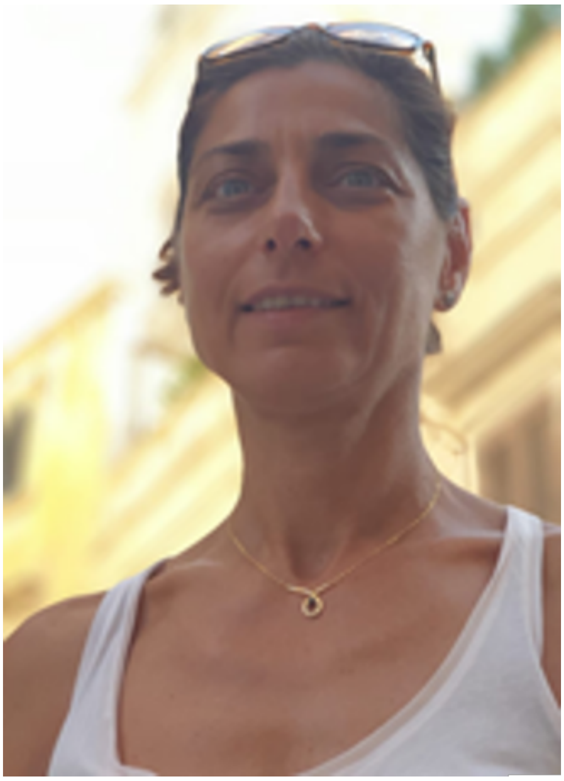

Supplement: Supplementary file 1 — Supplementary figures and tables. [file thnov10p7034s1.pdf]
